# The c-Myc/TBX3 Axis Promotes Cellular Transformation of Sarcoma-Initiating Cells

**DOI:** 10.3389/fonc.2021.801691

**Published:** 2022-01-25

**Authors:** Victoria Damerell, Melvin Anyasi Ambele, Shanel Salisbury, Alexis Neumann-Mufweba, Chrisna Durandt, Michael Sean Pepper, Sharon Prince

**Affiliations:** ^1^Division of Cell Biology, Department of Human Biology, Faculty of Health Sciences, University of Cape Town, Cape Town, South Africa; ^2^Department of Immunology and SAMRC Extramural Unit for Stem Research and Therapy, Faculty of Health Sciences, Institute for Cellular and Molecular Medicine, University of Pretoria, Pretoria, South Africa; ^3^Department of Oral Pathology and Oral Biology, School of Dentistry, Faculty of Health Sciences, University of Pretoria, Pretoria, South Africa

**Keywords:** mesenchymal stromal/stem cells (MSCs), sarcoma, c-Myc, TBX3, oncogene, tumorigenesis

## Abstract

Sarcomas are highly aggressive cancers of mesenchymal origin whose clinical management is highly complex. This is partly due to a lack of understanding of the molecular mechanisms underpinning the transformation of mesenchymal stromal/stem cells (MSCs) which are presumed to be the sarcoma-initiating cells. c-Myc is amplified/overexpressed in a range of sarcomas where it has an established oncogenic role and there is evidence that it contributes to the malignant transformation of MSCs. T-box transcription factor 3 (TBX3) is upregulated by c-Myc in a host of sarcoma subtypes where it promotes proliferation, tumor formation, migration, and invasion. This study investigated whether TBX3 is a c-Myc target in human MSCs (hMSCs) and whether overexpressing TBX3 in hMSCs can phenocopy c-Myc overexpression to promote malignant transformation. Using siRNA, qRT-PCR, luciferase reporter and chromatin-immunoprecipitation assays, we show that c-Myc binds and directly activates TBX3 transcription in hMSCs at a conserved E-box motif. When hMSCs were engineered to stably overexpress TBX3 using lentiviral gene transfer and the resulting cells characterised in 2D and 3D, the overexpression of TBX3 was shown to promote self-renewal, bypass senescence, and enhance proliferation which corresponded with increased levels of cell cycle progression markers (cyclin A, cyclin B1, CDK2) and downregulation of the p14^ARF^/MDM2/p53 tumor suppressor pathway. Furthermore, TBX3 promoted the migratory and invasive ability of hMSCs which associated with increased levels of markers of migration (Vimentin, SLUG, SNAIL, TWIST1) and invasion (MMP2, MMP9). Transcriptomic analysis revealed that genes upregulated upon TBX3 overexpression overlapped with c-myc targets, were involved in cell cycle progression, and were associated with sarcomagenesis. Together, the data described indicate that the c-Myc/TBX3 oncogenic molecular pathway may be a key mechanism that transforms hMSCs into sarcomas.

## Background

Sarcomas comprise a heterogenous group of neoplasms that are derived from mesenchymal tissues such as bone, cartilage, muscle, adipose and fibrous connective tissues. While relatively rare in adults (1% of all cancers), they account for >20% of all pediatric malignancies and in children and young adults they represent some of the most aggressive cancers (1). The clinical management of sarcomas remains uboptimal which is, in part, due to late diagnosis that results from a lack of specific symptoms and frequent misdiagnosis, resistance to current treatment modalities, local recurrence and metastases (1). The cells which give rise to sarcomas are still under debate, but growing evidence suggests that mesenchymal stromal/stem cells (MSCs), and in some instances mesenchymal progenitor cells, may be sarcoma-initiating cells (1–4). However, very little is known about the factors that drive their malignant transformation. This is important to elucidate because it will allow the identification of more reliable diagnostic markers and target-specific therapies.

At a molecular genetics level, whereas 15-20% of sarcomas have specific genetic alterations and relatively simple karyotypes with translocations resulting in oncogenic fusion proteins, two-thirds of sarcomas have complex karyotypes (1). Sarcomas with simple karyotypes include Ewing’s sarcoma, alveolar rhabdomyosarcoma and synovial sarcoma and sarcomas with a complex karyotype include osteosarcoma, liposarcoma and chondrosarcoma (other than myxoid) (1). While the oncogenic fusion proteins that drive sarcomas with a simple karyotype are reasonably well described, the factors that drive sarcomas with a complex karyotype are poorly understood. The transcription factor c-Myc is amplified/upregulated in several sarcomas with complex genetics including leiomyosarcoma, osteosarcoma, liposarcoma, chondrosarcoma and rhabdomyosarcoma, and this has been associated with cancer aggressiveness and poor clinical outcome (1, 5–11). Furthermore, c-Myc contributes to sarcomagenesis by promoting cancer stem cell expansion and by functioning as a pro-proliferative and pro-migratory factor. For example, in fibrosarcoma cells, c-Myc promotes stem-cell-like properties by binding and activating the *ABCG2* promoter and in rhabdomyosarcoma and Ewing’s sarcoma cells respectively it promotes proliferation by activating *MALAT1* and repressing *p21^CIP1^* (12–14). Importantly, the T-box transcription factor *TBX3* is upregulated in sarcoma cell lines and patient-derived sarcoma tissues including fibrosarcomas, chondrosarcomas, liposarcomas and rhabdomyosarcomas, and c-Myc transcriptionally activates *TBX3* to promote transition through S-phase in rhabdomyosarcoma and chondrosarcoma cells (15–17). TBX3 was shown to promote proliferation, tumor formation, migration, and invasion of chondrosarcoma, liposarcoma and rhabdomyosarcoma cells (15, 17).

Together the above results suggest that TBX3 is a key mediator of c-Myc induced sarcomagenesis and that c-Myc/TBX3 may be an important axis that drives MSCs into a subset of sarcomas. Indeed, various studies in murine and human MSCs (hMSCs) have already demonstrated that c-Myc may be key to their transformation (18–21). For example, c-Myc overexpression in combination with RB knockdown in hMSCs led to osteosarcoma formation *in vivo* (18). Shimizu et al. demonstrated that c-Myc overexpression alone was sufficient to transform mouse bone marrow-derived MSCs into osteosarcoma, and the process was substantially accelerated when the cell cycle control locus Ink4a-Arf was lost (19). In this regard, it is important to note that human TBX3 negatively regulates the INK4A-ARF locus (22, 23), and an inverse correlation between TBX3 and p14^ARF^ was observed in chondrosarcomas and fibrosarcomas (17).

We hypothesize that the c-Myc/TBX3 axis is important in sarcomagenesis and that TBX3 overexpression will drive hMSCs into sarcomas. Here we test this by performing a series of experiments in 2D- and 3D-cell culture models and also undertake a transcriptomic analysis. We show that TBX3 is transcriptionally activated by c-Myc in hMSCs and that overexpressing TBX3 in hMSCs promotes stemness, proliferation, migration, and invasion. Collectively our data provide evidence that TBX3 functions downstream of c-Myc in the transformation of hMSCs into sarcomas.

## Materials And Methods

### Cell Culture

Human adipose-derived MSCs from three different donors (hMSC lines 1-3) were isolated, characterized and expanded as previously described (24) and their identity was confirmed by flow cytometry using a panel of MSC markers CD73, CD90, CD44, CD36, CD45 and CD105 (**Supplementary Figure 1**). Approval was obtained from the Research Ethics Committee of the Faculty of Health Sciences, University of Pretoria (protocol number 218/2010) and written informed consent was obtained prior to lipoaspirate harvesting from healthy donors undergoing routine plastic or reconstructive surgery procedures. SW1353 chondrosarcoma cells (HTB-94) and SW872 liposarcoma cells (HTB-92) were obtained from ATCC. SaOS-2 (ATCC HTB-85) osteosarcoma cells were a gift from Abe Kasonga (University of Pretoria). Embryonic kidney HEK293FT cells were obtained from Thermo Fisher Scientific, USA. Cell cultures were maintained under standard culture conditions (37°C, 5% CO_2_, 65% humidity) in Dulbecco’s Modified Eagle Medium GlutaMax™ culture medium (DMEM, Gibco, Life Technologies/Thermo Fisher Scientific, USA) supplemented with 10-20% fetal bovine serum (FBS, Gibco, USA) and 1% penicillin and streptomycin (pen/strep, Gibco, USA). HEK293FT cells stably express the SV40 large T antigen and the neomycin resistance gene and were cultured with 500 μg/ml geneticin (G-418). At 80% confluence, hMSC cultures were passaged using 0.25% Trypsin/EDTA (Gibco, USA) and re-seeded at a density of 5000 cells/cm^2^. All experiments were performed with hMSCs at passages lower than 20.

### Immunophenotype

Immunophenotype analysis of hMSC lines 1-3 was performed as described previously (25). The hMSCs were positive for CD73, CD90, and CD105 and negative for CD45.

### RNA Interference

MSCs were plated at a density of 5000 cells/cm^2^ in a 24-well plate and transfected at 60% confluency with either 50 nM siTBX3 #1 (SI03100426, Qiagen, USA), 100 nM siTBX3 #2 (SI00083503, Qiagen, USA), 50 nM sic-Myc #1 (SI00300902, Qiagen, USA), 50 nM sic-Myc #2 (SI02662611, Qiagen, USA) or 50-100 nM control (non-silencing, siCtrl) (1027310, Qiagen, USA). Cells were transfected using HiPerFect^®^ transfection reagent (Qiagen, USA) according to manufacturer’s instructions.

### Plasmids

Lentiviral plasmids pCDH-Empty, pCDH-FLAG-TBX3 and envelope (p.VSV.G) and packaging (psPAX8) expression plasmids were kind gifts from Li Zhao (26). pCDH-FLAG-c-Myc was purchased from Addgene (plasmid # 102626; http://n2t.net/addgene:102626; RRID : Addgene_102626, a gift from Hening Lin) (27) and the FLAG-c-Myc construct was kindly provided by Professor Lüscher from Institut für Biochemie, Germany (28). The human TBX3 promoter luciferase reporter constructs were as previously described (16).

### Lentivirus Production and Transduction of hMSCs

Lentivirus was generated by co-transfecting pCDH-FLAG-TBX3 or pCDH-FLAG-c-Myc with p.VSV.G and psPAX8 plasmids into HEK293FT cells using Lipofectamine LTX transfection reagent (Invitrogen Life Technology, USA) according to manufacturer’s instructions. The empty pCDH-Empty vector (EV) with no insert was used as a control. Viral supernatant was harvested at 48 and 72 h post-transfection and concentrated by ultracentrifugation. Viral titer was determined using limiting-dilution colony formation titering assay. Using polybrene infection reagent (8 μg/mL, Merck, Germany), hMSC line 1 was transduced with viral supernatant [multiplicity of infection (MOI) of 0.3] to achieve TBX3/c-Myc overexpression and subjected to 0.5 μg/ml puromycin (Sigma, USA) for 16 - 18 days until colonies formed. Single cells were grown into colonies and sub-cultured into 24-well plates. The efficacy of stable TBX3 overexpression was confirmed by qRT-PCR, western blotting, and immunofluorescence.

### Western Blot Analysis

Total protein lysates were prepared as described previously (29). Primary antibodies used were: rabbit polyclonal anti-TBX3 (1:1000; ab99302; Abcam, Cambridge, UK), mouse monoclonal anti-β-actin (1:3000; sc-47778), mouse monoclonal anti-NANOG (1:500; sc-374001), rabbit polyclonal anti-cyclin A (1:1000; sc-751), mouse monoclonal anti-CDK2 (1:500; sc-6248), mouse monoclonal anti-MDM2 (1:500; sc-965), rabbit polyclonal anti-p16 (1:250; sc-759), rabbit polyclonal anti-p19/p14 (1:250), mouse monoclonal anti-p53 (1:500; sc-126), mouse monoclonal anti-SNAI (1:500; sc271977), mouse monoclonal anti-SLUG (1:500; sc-166476), mouse monoclonal anti-TWIST1 (1:500; sc-81417) from Santa Cruz Biotechnology Inc (Texas, USA); mouse monoclonal anti-MMP9 (1:1000; 4A3 NBP2-13173), mouse monoclonal anti-MMP2 (1:500; 8B4 NB200-114) from Novus Biotechnologicals (Colorado, USA); mouse monoclonal anti-cyclin B1 (1:1000; V152), rabbit monoclonal c-Myc (1:1000; D84C12) and rabbit polyclonal anti-vimentin (1:1000, R28) from Cell Signaling Technology (Massachusetts, USA); rabbit polyclonal anti-p38 (1:5000; M0800), from Sigma-Aldrich (Missouri, USA). Secondary antibodies were used at 1:5000 dilution and included horseradish peroxidase (HRP)-conjugated goat anti-rabbit (Biorad, USA), goat anti-mouse (Biorad, USA). Densitometry was performed using the image analysis software Fiji (Version 2.0.0-rc-68/1.52e) (30) and normalised to the appropriate loading control. All blots are representative of at least two independent repeats.

### Quantitative Real-Time PCR

Total RNA was extracted using the High Pure RNA Isolation Kit (Roche, Germany) according to the manufacturer’s instructions, and reverse transcription was performed using 500 ng RNA and the InProm-II™ reverse transcription system (Promega A3800, USA) according to the manufacturer’s instructions. The reactions were carried out using 1 μL cDNA, 2x SYBR green master mix (Applied Biosystems, USA) and primers to amplify the human TBX3 (QT00022484, Qiagen, USA), GUSB (QT00046046, Qiagen, USA), c-Myc (F: CTGAGACAGATCAGCAACAACC; R: TTGTGTGTTCGCCTCTTGAC, Integrated DNA Technologies, USA), p16^INK4a^ (F: GTGGACCTGGCTGAGGAG; R: CTTTCAATCGGGGATGTCTG, Integrated DNA Technologies, USA). qRT-PCR was performed using the Light Cycler^®^ 2.0 system (Roche, Switzerland) and the following parameters: denaturation for 15 min at 95°C and combined annealing and extension for 40 cycles at 60°C for 1 min. Samples were prepared in triplicate and non-template controls were run to detect contamination or nonspecific amplification. The 2^−ΔΔCt^ method was employed to analyze results, and relative mRNA expression levels of c-Myc, TBX3, and p16^INK4a^ were normalized to mRNA levels of glucuronidase-beta (GUSB).

### Immunofluorescence

Immunofluorescence was performed as previously described (16). Briefly, cells were incubated with rabbit polyclonal anti-TBX3 antibody (1:100 dilution; ab99302, Abcam, USA) at 4°C overnight. Subsequently, cells were incubated with fluor-conjugated secondary Cy3 donkey anti-rabbit IgG (1:1000 dilution; Jackson ImmunoResearch Laboratories, Inc., USA). To visualize nuclei, cells were incubated with 1 mg/mL Hoechst 33258 (Thermofisher Scientific, South Africa). Fluorescent cells were viewed using an Axiovert confocal microscope (Zeiss, USA).

### Proliferation Assay

Short-term growth was measured as described previously (29). hMSCs were seeded in triplicate at a density of 5000 cells/cm^2^ in 24-well plates. Cells were collected by trypsinization and counted on a haemocytometer at 2–3 days intervals. As an alternative assay for proliferation, cell viability was determined using the 3-(4,5-dimethylthiazol-2-yl)-2,5-diphenyltetrazolium bromide (MTT) Cell Proliferation Kit (Roche, Switzerland). Cells were seeded in quadruplicate in 96-well plates (5000 cells/cm^2^) and cell viability determined over 5 days according to the manufacturer’s instructions. Absorbance (595 nm) was measured using GloMax^®^ plate reader (Promega, USA) and the absorbance of the medium only control was subtracted from the samples.

### *In Vitro* Cell Migration Assays

Cell migration was measured using a two-dimensional (2D) *in vitro* scratch motility assay as previously described (31). The wound areas were measured over time and calculated using the image analysis software Fiji (Version 2.0.0-rc-68/1.52e).

### Colony Formation Unit Assay

Cells were seeded at 100 cells/35 mm dish in triplicate and the culture medium was changed every 3 days. After 18 days, the colonies were washed twice with 1x PBS, fixed with ice-cold methanol for 15 min, stained with 1% crystal violet dye (C3886, Sigma, USA) for 1 h, and washed once with 1x PBS and twice with tap water to remove excess crystal violet. Photographic images were captured to count the colonies.

### Senescence-Associated β-Galactosidase Staining

Cells were seeded at 5000 cells/cm^2^ in 6-well plates and 48 h later they were stained using a Senescence β-Galactosidase Staining Kit (#9860, Cell Signaling Technology, USA) following the manufacturer’s protocol. Images were taken using an EVOS M5000 Imaging System microscope (Thermo Fisher Scientific, USA).

### Three-Dimensional Spheroid Formation

To establish spheroids that finally consist of 5000 cells/spheroid, cells were suspended in DMEM at 50,000 cells/mL and 100 μL of this suspension was plated per well in a 96-well plate coated with 1.2% agarose (SeaKem LE Agarose 50004, Lonza, USA) to prevent cell adhesion. Cells were incubated for 4 days to allow for compact spheroid formation before proceeding with the experiments described below.

### Spheroid Growth Assay

Images (EVOS XL AMEX Core Imaging System) were taken (t=Day 1) and cell growth was monitored over five days (t=Day 5). The area of spheroid core and periphery was measured using the image analysis software Fiji (Version 2.0.0-rc-68/1.52e) and the ratio of spheroid core to periphery was calculated.

### Calcein AM Staining of Spheroids

The following three fluorescent stains were used to determine cell viability: calcein AM (1 mg/mL, C1430, Invitrogen, Massachusetts, USA), propidium iodide (PI) (1 mg/mL), and 4′,6-diamidino-2-phenylindole (DAPI) (500 µg/mL, Thermo Fisher Scientific, USA). A 2x staining solution in medium was prepared by mixing calcein AM, PI, and DAPI to working concentrations of 2 µM, 8 µg/mL, and 20 µg/mL, respectively. Spheroids were stained by removing 50 µL of medium and replaced with 50 µL of the 2x staining solution, for final well concentrations of 1 µM, 4 µg/mL, and 10 µg/mL, respectively. Plates were then incubated at 37°C and 5% CO_2_ for 60 min and imaged using an EVOS M5000 Imaging System microscope (Thermo Fisher Scientific, USA). Calcein AM staining intensity was measured using the image analysis software Fiji (Version 2.0.0-rc-68/1.52e) and calculated relative to the spheroid area.

### Spheroid Invasion Assay

Spheroids were formed as described earlier and each spheroid was then transferred to individual wells of a 96-well plate where they were embedded in 70 μL of 1.5 mg/mL collagen type I rat tail matrix (Gibco, A1048301, Thermo Fisher Scientific, USA) and covered with 100 μL of complete media. Cell invasion was monitored using an EVOS M5000 Imaging System microscope (Thermo Fisher Scientific, USA) and images were taken after 0, 24, and 48 h. The invasive area was determined by calculating the difference between the final area (t=24, 48 h) and the initial area (t=0 h) using the image analysis software Fiji (Version 2.0.0-rc-68/1.52e), and data were normalized to control cells. Three independent experiments including four replicates for each condition were performed.

### Anchorage-Independent Assay

Dishes (35 mm) were layered first with 0.6% agar prepared in cell culture medium followed by 0.3% agar prepared in cell culture medium containing 5000 cells/dish. The dishes were incubated for 12 weeks, and colonies were imaged using an EVOS M5000 Imaging System microscope (Thermo Fisher Scientific, USA). Relative colony area was measured using the image analysis software Fiji (Version 2.0.0-rc-68/1.52e), and data were normalized to control cells. Two independent experiments including three replicates for each condition were performed.

### Chromatin-Immunoprecipitation (ChIP) Assay

ChIP assays were performed as previously described (32). Briefly, hMSCs were fixed in 1% formaldehyde, the chromatin extracted and sonicated to obtain DNA fragments of between 300 and 500 bp. Protein-bound DNA was immunoprecipitated using antibodies against c-Myc (8 μg; E5Q6W, Cell Signaling, USA) or normal rabbit IgG (8 μg; sc-2729, Santa Cruz Biotechnology Inc, USA). The DNA to which c-Myc bound was purified using the phenol-chloroform extraction method and precipitated DNA was analysed by qRT-PCR using primers spanning TBX3 E-box -1936; -1789 (forward, 5′- GGGAATTCTCAACGCTGG -3′; reverse, 5′ CAGCACAGGCCTCTCTCG 3′); E-box -1210 (forward, 5′- GAG AAG ATA CCA GGC TGG C-3′; reverse, 5′ CAT ATT CCA CCT GGA TGT GGG3′); E-box -701 (forward, 5′- GAG ACC AGC ACC GAG ACA C-3′; reverse, 5′ GGC CAC TCG GTT CTA CAA AAG-3′) and GAPDH (forward, 5′-GAAGGCTGGGGCTCATTT-3′; reverse, 5′-CAGGAGGCATTGCTGATGAT-3’). Crossing values (Ct) of c-Myc and IgG precipitated DNA were adjusted by normalizing against the Ct value of 1% of input DNA and the ΔΔCt method was used to determine fold enrichment using the following equation: 2^-(ΔCt1−ΔCt2)^ (ΔCt1 = c-Myc, ΔCt2 = IgG).

### Dual Luciferase Reporter Assay

For luciferase assays, hMSCs were plated in 12-well plates at a density of 5000 cells/cm^2^. The next day, 500 ng of the TBX3 luciferase reporter plasmid and increasing amounts (50, 100 and 200 ng) of the FLAG-c-Myc expression vector or empty vector were transfected using Lipofectamine LTX (Invitrogen Life Technology, USA) according to manufacturer’s instructions. The vector pRL-TK, which contains the thymidine kinase promoter driving expression of a renilla reporter, was included as an internal control for transfection efficiency (20 ng per transfection). Cells were lysed 48 h after transfection, and firefly and renilla luciferase activities were determined using the Dual-Luciferase Reporter Assay System (Promega, USA) according to the manufacturer’s instructions and measured using a Luminoskan Ascent luminometer (Thermo Labsystems, USA). Extracts were subjected to western blot analysis to confirm expression of transfected c-Myc protein.

### Transcriptomic Analysis

Total RNA was extracted from EV (EV #1, EV #2) and TBX3 (TBX3 #1, TBX3 #2) hMSCs (three biological replicates per clone) using the High Pure RNA Isolation Kit (Roche, Germany) according to the manufacturer’s instructions and was used for microarray gene expression analysis on the human Affymetrix Clariom S array. Briefly, a total of 5 ng RNA from each sample was used to synthesize first strand cDNA, followed by 3’ adaptor and double stranded cDNA (ds-cDNA) using the Clariom S array (IVT) Pico kit according to the manufacturer’s protocol. The ds-cDNA was used to synthesize cRNA by *in vitro* transcription for 14 h at 40°C and the cRNA was purified using purification beads on a magnetic stand. A total of 20 µg purified cRNA was used for 2nd-cycle ds-cDNA synthesis followed by RNA hydrolysis and ds-cDNA purification as described above. Thereafter, 5.5 µg of each sample was used for fragmentation and labelling, followed by hybridization on the Clariom S array in an Affymetrix GeneChip^®^ Hybridisation Oven-645 rotating at 60 rpm at 45°C for 16 h. Hybridised chips were washed and stained using GeneChip™ Hybridization, Wash and Stain Kit in an Affymetrix GeneChip^®^ Fluidics Station-450Dx before scanning using an Affymetrix GeneChip^®^ Scanner-7G. From each scanned chip, the Affymetrix system generates a CEL file which has intensity values for all probes present on it. The CEL files were imported into the Affymetrix Transcriptome Analysis Console (TAC) v4.0.2 software for differential gene expression analysis. Genes considered to be differentially expressed (DEGs) between EV and TBX3 hMSCs were those with fold-change (FC) values ≥ 2 or ≤ −2 and ANOVA adjusted-p value < 0.05. The DEGs between EV and TBX3 hMSCs were further visualized on a volcano plot and hierarchical clustering using TAC 4.0.2 software. The microarray data files of this study have been deposited in NCBI GEO (Gene Expression Omnibus) with assigned GEO accession number GSE183848.

### Gene Ontology, KEGG, and GSEA Analyses

Gene ontology (GO) biological process and Kyoto Encyclopedia of Genes and Genomes (KEGG) pathway enrichment analyses were performed using g:Profiler (version e104_eg51_p15_3922dba) with the Benjamini-Hochberg FDR method applying a significance threshold of 0.05 (33). To identify gene signatures or pathways enriched in TBX3 hMSCs, gene set enrichment analysis (GSEA) was performed with the GSEA program (v.4.1.0) (34, 35). The Broad Molecular Signatures Database (MSigDB v7.4) set H (hallmark gene sets, 50 gene sets, h.all.v7.4.symbols.gmt) was selected as the reference. The permutation number was set to 1,000 and cut-off values of nominal p  <  0.05 and FDR  <  0.25 were selected.

### Statistical Analysis

Statistical significance was determined using a student’s t-test (Excel, Microsoft, Redmond, WA, USA). Significance was accepted at *p < 0.05, **p < 0.01, ***p < 0.001. Unless otherwise stated, all data were obtained from at least three independent experimental repeats with error bars representing standard error of the mean (SEM). Graphs were generated using GraphPad Prism software 6.0 (GraphPad Prism software, USA).

## Results

### The TBX3 Promoter Is Transcriptionally Activated by c-Myc in hMSCs

**Figure 1:**
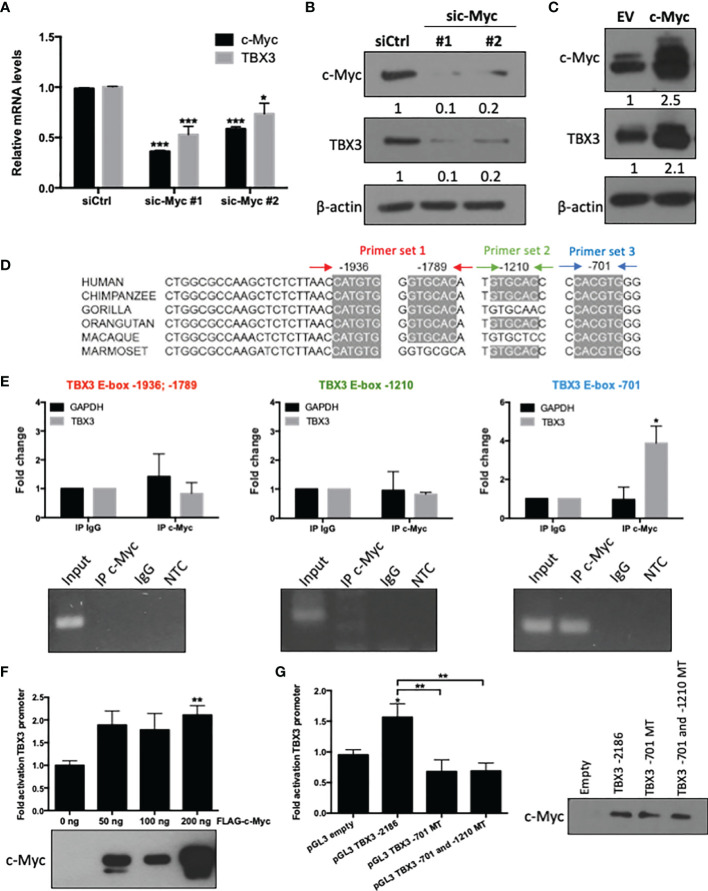
The TBX3 promoter is transcriptionally activated by c-Myc in hMSCs. hMSCs were transiently transfected with 50 nM sic-Myc (#1, #2) or siControl (siCtrl) for 48 h and levels of c-Myc and TBX3 **(A)** mRNA and **(B)** protein were assessed using qRT-PCR and western blotting respectively. **(C)** Western blot analysis show levels of c-Myc and TBX3 in hMSCs transduced with empty vector (EV) or a c-Myc expression construct using lentiviral gene transfer. For western blotting, β-actin was used as a loading control. Densitometry readings were obtained using Fiji and protein expression levels are represented as a ratio of protein of interest/β-actin normalized to siCtrl or EV. **(D)** Representation of four highly conserved c-Myc consensus E-box motifs within the TBX3 promoter. **(E)** Chromatin immunoprecipitation (ChIP) assays were performed using DNA immunoprecipitated from hMSCs with an antibody against c-Myc or IgG (negative control). Upper panel (graphs): Immunoprecipitated DNA was assayed by qRT-PCR with primers spanning the E-box sites as indicated in **(D)**. Primers specific to GAPDH were included as a negative control. Lower panel (agarose gel electrophoresis): qRT-PCR products of Input, IP c-Myc, IgG and non-template control (NTC). **(F)** Luciferase assays of hMSCs co-transfected with 500 ng of TBX3 and 50–200 ng of FLAG-c-Myc expression construct. Total amount of plasmid DNA transfected was held constant using the corresponding empty vector. **(G)** Luciferase assays of cells co-transfected with 200 ng of c-Myc expression construct and either 500 ng of TBX3 promoter reporter or with TBX3 promoter reporter with mutated (MT) E-box motifs. **(F, G)** The pRL-TK renilla luciferase reporter plasmid was used to control for transfection efficiency. Data were normalized against renilla values and fold activation of the TBX3 promoter was calculated relative to the empty vector control. Western blots show expression of transfected c-Myc. Student’s t-test was used to compare between groups, *p < 0.05; **p < 0.01; ***p < 0.001; error bars represent mean ± SEM (n=3, for all panels).

To explore whether the c-Myc/TBX3 axis exists in hMSCs we first investigated whether, as is the case in sarcomas, c-Myc transcriptionally activates TBX3. To address this, we determined whether altering c-Myc levels resulted in a corresponding change in TBX3 levels in hMSCs. Indeed, we show that depleting c-Myc by siRNA (sic-Myc #1 or sic-Myc #2) resulted in decreased levels of TBX3 mRNA (**Figure 1A**) and protein (**Figure 1B**) and overexpressing c-Myc resulted in increased TBX3 levels (**Figure 1C**). These results show that c-Myc is indeed upstream of TBX3 in hMSCs. The TBX3 promoter has four highly conserved c-Myc consensus E-box sites at positions -1936, -1789, -1210 and -701 base pairs (bps) (**Figure 1D**). ChIP assays showed that while c-Myc did not occupy the regions of the TBX3 promoter containing E-box sites at -1936; -1789 and -1210 bps, its binding to the canonical E-box site at -701 (CACGTG) was enhanced by 3.88-fold (**Figure 1E**). Luciferase reporter assays confirmed that 200 ng c-Myc significantly activated the TBX3 promoter in hMSCs (**Figure 1F**), and importantly, mutation of the E-box at -701 bp abrogated this activation (**Figure 1G**). Western blot analysis of lysates from luciferase assays confirmed expression of transfected c-Myc in these experiments (**Figures 1F, G****)**. Together these results confirm that, in hMSCs, the TBX3 promoter is transcriptionally activated by c-Myc through the -701 bp E-box motif.

### TBX3 Contributes to hMSC Proliferation and Migration

**Figure 2:**
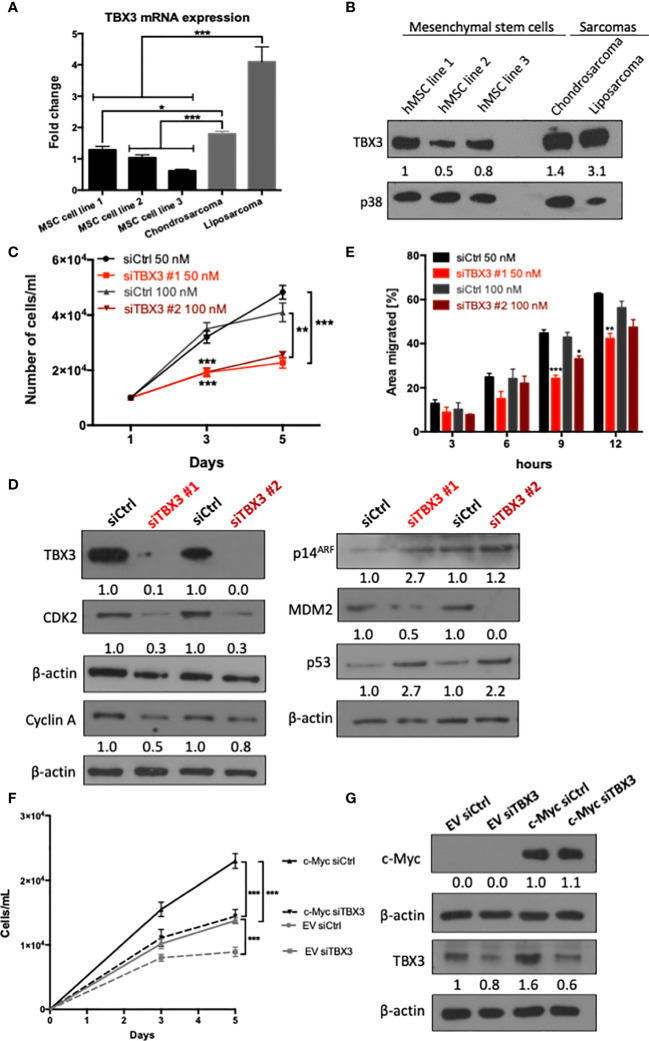
TBX3 contributes to hMSC proliferation and migration. **(A)** qRT-PCR analysis of TBX3 mRNA expression in adipose-derived hMSCs from three different donors (MSC cell lines 1-3) as well as chondrosarcoma (SW1353) and liposarcoma (SW872) cells. **(B)** Western blot analysis of TBX3 protein expression in the cells described in **(A)**. p38 was used as a loading control. **(C–E)** hMSCs were transiently transfected with siTBX3 or siControl (siCtrl) and **(C)** Growth curve assays were performed over a 5-day period; **(D)** Western blotting of protein harvested from cells in **(C)** on day 5 with antibodies to TBX3, CDK2, cyclin A, p14^ARF^, MDM2, and p53; and **(E)** 2D-Scratch motility assays were performed where a linear wound was made on confluent transiently transfected hMSCs and distance migrated was measured at 3, 6, 9 and 12 h. **(F, G)** hMSCs were stably transduced with EV or a c-Myc lentiviral expression construct and transiently transfected with siTBX3 or siControl (siCtrl). **(F)** Growth curve assays were performed over a 5-day period; **(G)** Western blotting of protein harvested from cells in **(F)** on day 5 with antibodies to c-Myc and TBX3. For western blotting, β-actin was used as a loading control and densitometry readings were obtained using Fiji and protein expression levels are represented as a ratio of protein of interest/p38 or protein of interest/β-actin normalized to hMSC line 1 or siCtrl respectively. Student’s t-test was used to compare between groups, *p < 0.05; **p < 0.01; ***p < 0.001; error bars represent mean ± SEM (n=3, for all panels).

If the upregulation of TBX3 by c-Myc is a key molecular mechanism involved in transforming hMSCs to sarcomas, then one would expect that parental hMSCs will have lower levels of TBX3 than TBX3-driven sarcomas. Therefore, before we engineered hMSCs to overexpress TBX3, we compared the status of TBX3 mRNA and protein in three adipose-derived hMSC cell lines (hMSC line 1-3) as well as chondrosarcoma (SW1353) and liposarcoma (SW872) cells by qRT-PCR and western blotting respectively. Our results show that TBX3 mRNA and protein levels were lower in hMSCs compared to chondro- and liposarcoma cells (**Figures 2A, B****)**. We speculated that endogenous TBX3 contributes to hMSC proliferation and migration and that during the malignant transformation of hMSCs, TBX3 is upregulated and promotes these as well as other oncogenic processes. Indeed, transiently depleting TBX3 by siRNA (siTBX3) significantly inhibited hMSC proliferation (**Figure 2C**), decreased levels of the cell cycle progression markers cyclin A and CDK2, increased the negative cell cycle regulators p14^ARF^ and p53, and decreased levels of the negative p53 regulator, MDM2 (**Figure 2D**). Furthermore, depleting TBX3 retarded the migratory ability of hMSCs (**Figure 2E**). We next investigated whether TBX3 promotes hMSC proliferation downstream of c-Myc. To this end, TBX3 was depleted in hMSCs stably overexpressing c-Myc (c-Myc siTBX3) or hMSC empty control (EV siTBX3) cells and the impact on cell proliferation measured. Growth curve assays show that overexpressing c-Myc (c-Myc siCtrl) significantly enhanced hMSC proliferation and depleting TBX3 (EV siTBX3) inhibited their proliferation (**Figures 2F, G****)**. Importantly, depleting TBX3 abrogated the pro-proliferative activity of c-Myc (c-Myc siTBX3). Together, these data suggest that the upregulation of TBX3 by c-Myc is a key downstream event in mediating the ability of c-Myc to promote hMSC proliferation.

### Establishment of hMSC Lines Stably Overexpressing TBX3

**Figure 3:**
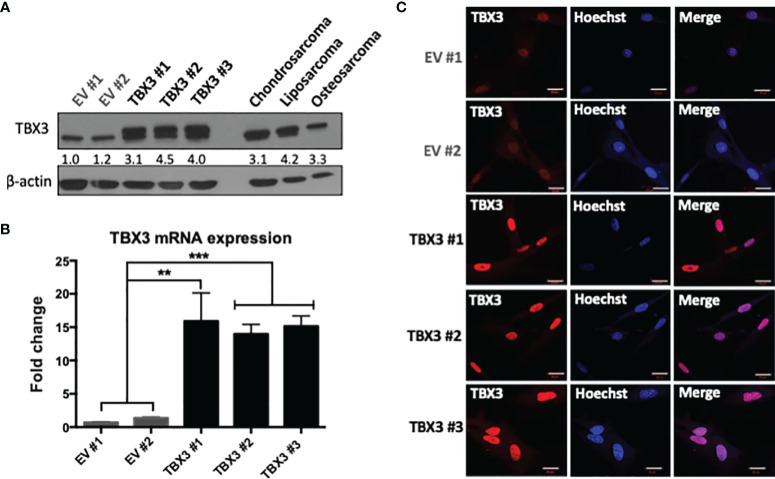
Establishment of stable TBX3 overexpressing hMSCs. **(A)** Western blot analysis shows levels of TBX3 in EV and TBX3 hMSCs, chondrosarcoma (SW1353), liposarcoma (SW872) and osteosarcoma (SaOS-2) cells. β-actin was used as a loading control. Densitometry readings were obtained using Fiji and protein expression of total TBX3 levels (endogenous + FLAG-tagged) are represented as a ratio of protein of interest/β-actin normalized to EV #1. **(B)** qRT-PCR analysis shows levels of TBX3 mRNA in EV and TBX3 hMSCs. Student’s t-test was used to compare between groups, **p < 0.01; ***p < 0.001; error bars represent mean ± SEM. **(C)** Representative confocal immunofluorescence images (630X; Carl Zeiss LSM 510, scale bars = 20 µm) of EV and TBX3 hMSCs. TBX3 was detected with a fluorophore conjugated Cy3 secondary antibody. Cells were co-stained with Hoechst to determine nuclei location and 20 fields of view were visualized (n=3, for all panels).

To determine if TBX3 overexpression in hMSCs can promote their transformation, we first established hMSCs that stably overexpress TBX3 using a lentiviral transduction system (**Supplementary Figure 2**). Briefly, hMSCs were transduced with lentiviruses delivering FLAG-TBX3 or Empty vector (EV) and resistance to the mammalian antibiotic puromycin enabled selection of successfully transduced cells. In total, 13 EV hMSC and 15 FLAG-TBX3 hMSC puromycin-resistant clones were obtained and they all tested positive for FLAG-TBX3 (data not shown). **Figures 3A** **–C** show EV hMSC (hereafter referred to as EV #1 and EV #2) and FLAG-TBX3 hMSC (hereafter referred to as TBX3 #1, TBX3 #2 and TBX3 #3) clones that were selected for further analyses. Compared to EV hMSCs, TBX3 hMSCs expressed significantly higher levels of TBX3 protein (**Figure 3A**), mRNA (**Figure 3B**) and nuclear localization (**Figure 3C**). Furthermore, TBX3 protein levels in the TBX3 clones were comparable to those in chondrosarcoma (SW1353), liposarcoma (SW872) and osteosarcoma (SaOS-2) cells (**Figure 3A**). The immunofluorescence results shown in **Figure 3C** confirm that TBX3 localizes to the nucleus and that the FLAG-tag therefore did not affect its localization and consequently its function as a transcription factor.

### TBX3 Promotes Stemness, Self-Renewal and Bypass of Senescence in hMSCs

**Figure 4:**
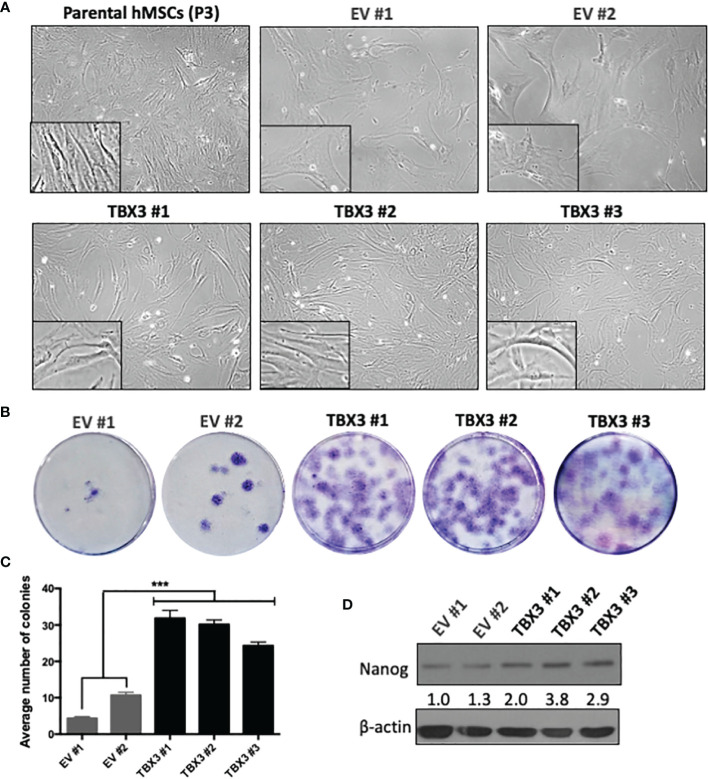
hMSCs stably overexpressing TBX3 maintain stemness. **(A)** Cell morphology of parental (early passage, P3) and late passage (P15) EV and TBX3 hMSCs. Representative light microscopy images (100X; EVOS XL AMEX Core Imaging System) are shown. **(B)** Colony formation unit assay of EV and TBX3 hMSCs. Cells were seeded at 100 cells in 35mm dishes, and colonies were stained using 1% Crystal violet and counted after 18 days. **(C)** Quantification of colony formation assay. Student’s t-test was used to compare between groups, ***p < 0.001; error bars represent mean ± SEM (n=3). **(D)** Western blot analysis of NANOG expression. β-actin was used as a loading control (n=2). It is important to note that TBX3 (**Figure 3A**) was detected on the same western blot, and therefore the blots for β-actin are the same. Densitometry readings were obtained using Fiji and protein expression levels are represented as a ratio of protein of interest/β-actin normalized to EV #1.

Stemness is a feature of cancer cells that drives tumor progression by enhancing self-renewal capacity, survival, proliferation, and metastasis (36). While expanding the EV and TBX3 hMSCs we noticed that with increasing *ex vivo* passages the EV hMSCs exhibited a flattened and senescent-like morphology but TBX3 hMSCs at the same passages maintained an elongated and spindle-shape morphology like early passage parental hMSCs (Passage 3) (**Figure 4A**). Furthermore, compared to EV hMSCs, TBX3 hMSCs had on average a 3.67-fold enhanced colony forming ability (**Figures 4B, C****)** and expressed higher levels of the stem cell marker NANOG (**Figure 4D**). Together, these findings suggest that overexpressing TBX3 in hMSCs promotes their stemness characteristics and prevents them from differentiating and undergoing senescence.

**Figure 5:**
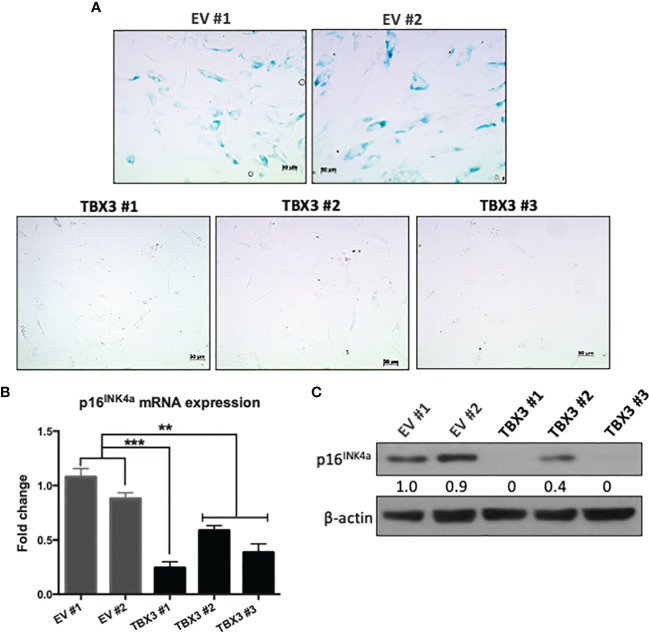
When overexpressed in hMSCs, TBX3 promotes bypass of senescence. **(A)** Senescence-Associated (SA)-β-Galactosidase staining of EV and TBX3 hMSCs. Representative brightfield microscopy images (200X; EVOS M5000 Imaging System; scale bars = 50 µm) are shown. **(B)** qRT-PCR and **(C)** Western blot analysis show *p16^INK4a^* mRNA and protein expression respectively in EV and TBX3 hMSCs. **(C)** β-actin was used as a loading control. Densitometry readings were obtained using Fiji and protein expression levels are represented as a ratio of protein of interest/β-actin normalized to EV #1. Student’s t-test was used to compare between groups, **p < 0.01; ***p < 0.001; error bars represent mean ± SEM (n=3, for all panels).

We next investigated whether, when overexpressed in hMSCs, TBX3 indeed functions as an anti-senescence factor. Our results show that, compared to EV hMSCs, TBX3 hMSCs exhibited substantially less SA-β-gal activity (**Figure 5A**) and expressed significantly lower levels of the senescence marker *p16^INK4a^* (**Figures 5B, C****)**. These results are important because for cancer cells to achieve a state of immortality, they need to escape cellular senescence.

### TBX3 Promotes hMSC Proliferation and Migration

**Figure 6:**
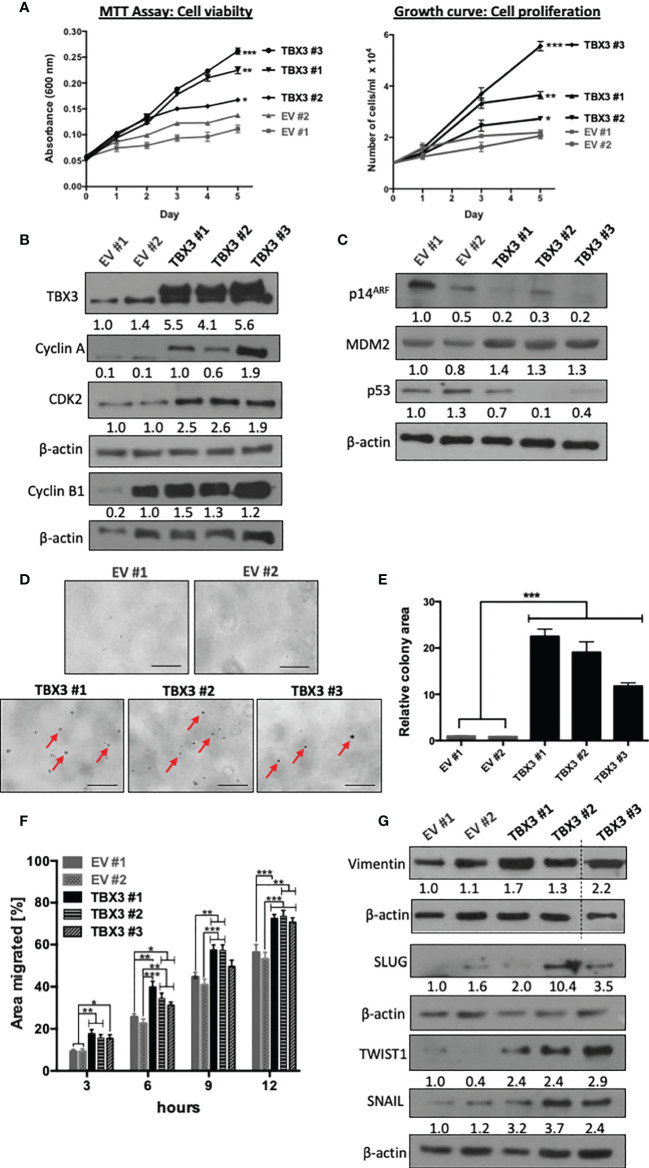
TBX3 promotes hMSC cell viability, anchorage-dependent and -independent cell proliferation and migration. **(A)** MTT Assay (left panel) and cell counting (right panel) of EV and TBX3 hMSCs was performed over a 5-day period. Student’s t-test was used to compare between groups, *p < 0.05; **p < 0.01; ***p < 0.001; error bars represent mean ± SEM (n=3). **(B, C)** Western blot analysis with antibodies to the indicated proteins (n=3). **(D, E)** Soft agar assay of EV and TBX3 hMSCs. Cells were seeded at 100 cells per 35 mm dish, and colony formation was measured over 3 months. **(D)** Representative microscopic images. Red arrows indicate TBX3 hMSC colonies (100X; EVOS M5000 Imaging System, scale bars = 250 µm). **(E)** Quantification of relative colony area using Fiji. Student’s t-test was used to compare between groups, ***p < 0.001; error bars represent mean ± SEM (n=2). **(F)** 2D-Scratch motility assays were performed where a linear wound was made on EV or TBX3 hMSC monolayers and distance migrated was measured at 3, 6, 9 and 12 h. Student’s t-test was used to compare between groups, *p < 0.05; **p < 0.01; ***p < 0.001; error bars represent mean ± SEM (n=3). **(G)** Western blot analysis with antibodies to the indicated proteins (n=2). **(B, C, G)** β-actin was used as a loading control. Densitometry readings were obtained using Fiji and protein expression levels are represented as a ratio of protein of interest/β-actin normalized to EV #1.

The ability to sustain persistent proliferation is one of the most fundamental traits of cancer cells (37). MTT and growth curve assays showed that TBX3 significantly promoted hMSC viability and proliferation (**Figure 6A**) respectively, which was associated with an increase in cyclin A, CDK2 and cyclin B1 (**Figure 6B**). Furthermore, TBX3 hMSC had a decrease in p14^ARF^, an increase in MDM2 (a negative regulator of p53), and a corresponding decrease in p53 levels (**Figure 6C**). These results are consistent with previous studies that showed that TBX3 promotes proliferation and immortalization by interfering with the p14^ARF^-MDM2-p53 pathway (17, 22, 38, 39). To form tumors as well as to migrate and metastasize, cancer cells have to exhibit anchorage-independent cell growth (40). Results from soft agar assays show that TBX3 hMSCs proliferated and formed colonies (indicated by red arrows) in the absence of a substrate whereas EV hMSCs remained as single cells (**Figures 6D, E****)**.

Cell migration and invasion of cancer cells into surrounding tissue and lymphatic and/or vascular systems is the initial step of tumor metastasis (41). To explore whether TBX3 promotes the migratory ability of hMSCs, we performed two-dimensional scratch motility assays and the results show that TBX3 hMSCs migrated significantly faster than EV hMSCs (**Figure 6F**). This was associated with an increase in the mesenchymal migration marker vimentin and the transcription factors SLUG, SNAIL and TWIST1 (**Figure 6G**).

### TBX3 Promotes 3D Spheroid Viability, Growth, and Invasion

**Figure 7:**
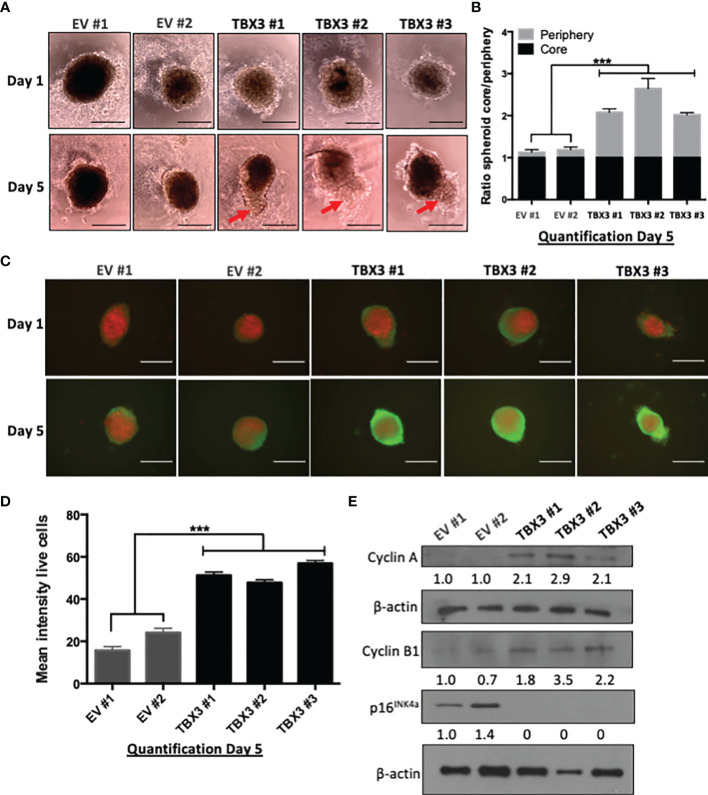
TBX3 promotes 3D spheroid viability and growth. **(A)** 3D spheroid growth assay of EV and TBX3 hMSCs. hMSCs were plated on 1.2% agarose to prevent cell adhesion and incubated for 96 h to allow for compact spheroid formation. The next day (Day 1) and on Day 5 spheroids were imaged (100X; EVOS XL AMEX Core Imaging System, scale bars = 200 µm). Red arrows indicate protrusions of the TBX3 hMSC spheroid periphery (proliferating cells). **(B)** Quantitative analysis of spheroid core/spheroid periphery ratio on Day 5 using Fiji. **(C)** Cell viability/proliferation was assessed by calcein AM (live cells; green) and propidium iodide (dead cells; red) staining. Immunofluorescence images (100X; EVOS XL AMEX Core Imaging System; scale bars = 250 µm) were taken at Day 1 and Day 5. **(D)** Quantitative analysis of mean intensity of live cells using Fiji. **(E)** Western blot analysis with antibodies to cyclin A, cyclin B1 and p16^INK4a^. β-actin was used as a loading control. Densitometry readings were obtained using Fiji and protein expression levels are represented as a ratio of protein of interest/β-actin normalized to EV #1. Student’s t-test was used to compare between groups, ***p < 0.001; error bars represent mean ± SEM (n=3, for all panels).

To test whether overexpression of TBX3 also promotes hMSC viability, proliferation, and invasion under more physiologically relevant conditions, we generated 3D spheroids of EV hMSCs and TBX3 hMSCs. 3D spheroid culture models are superior to 2D cell cultures because they better mimic the structure of the natural *in vivo* cell environment such as cell-cell and cell-extracellular matrix interactions (42). The periphery of TBX3 hMSC spheroids was significantly larger than that of the EV hMSC spheroids which is indicative of viable and proliferating cells (**Figures 7A, B****)**. Calcein AM staining for live cells (green) and propidium iodide staining for dead cells (red) confirmed that the cells on the periphery of the TBX3 hMSC spheroids are indeed more viable and proliferative than those on the periphery of the EV hMSC spheroids (**Figures 7C, D****)**. These results were accompanied by a downregulation of p16^INK4a^ and an increase in cyclin A and cyclin B1 (**Figure 7E**).

**Figure 8:**
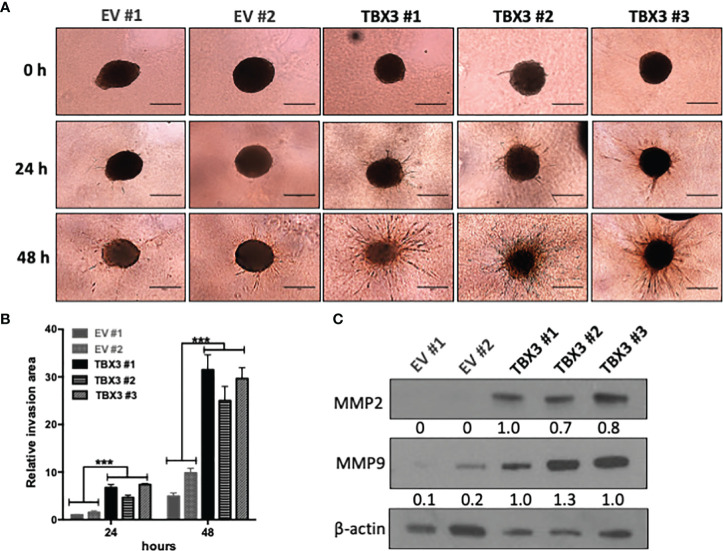
TBX3 promotes 3D spheroid invasion. **(A)** 3D spheroid invasion assay of EV and TBX3 hMSCs. Representative images of the 3D invading spheroids at 0, 24 and 48 h. (40X; EVOS M5000 Imaging System; scale bars = 600 µm). **(B)** Quantification of the invasive area. Student’s t-test was used to compare between groups, ***p < 0.001; error bars represent mean ± SEM (n=3). **(C)** Western blot analysis of MMP2 and MMP9 expression in 3D hMSC spheroids (n=2). For western blotting β-actin was used as a loading control. Densitometry readings were obtained using Fiji and protein expression levels are represented as a ratio of protein of interest/β-actin normalized to TBX3 #1.

We next investigated the ability of hMSC spheroids to invade collagen matrices which are organized 3D structures that mimic a tumor micro-region (43). Our results show that, compared to EV hMSC spheroids, TBX3 hMSC spheroids were significantly more invasive at 24 h and 48 h (**Figures 8A, B****)** which correlated with higher levels of the invasion markers MMP2 and MMP9 (**Figure 8C**).

### Effect of TBX3 Overexpression on Changes of hMSC Gene Expression

**Figure 9:**
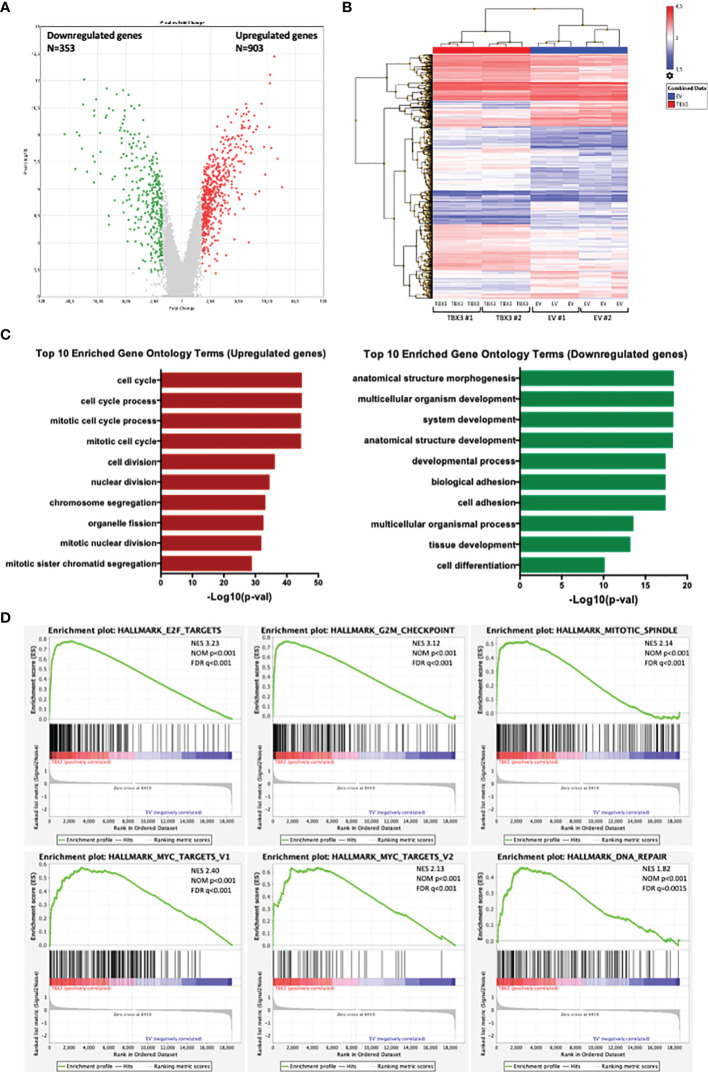
Analysis of differentially expressed genes (DEGs) in EV and TBX3 hMSCs. **(A)** Volcano plot. Red dots represent significantly upregulated genes (FC ≥ 2, p < 0.05); green dots represent significantly downregulated genes (FC ≤ −2, p < 0.05); grey dots represent DEGs below the level of significance. **(B)** Heat map showing hierarchical cluster analysis of significantly upregulated and downregulated genes (n=3). **(C)** Top 10 Gene ontology (Biological process) enrichment terms for upregulated (left) and downregulated (right) genes. **(D)** Gene set enrichment analysis (GSEA) of DEGs showing the top six significant (FDR < 0.25, NOM P-value < 0.05) enrichment terms of the hallmark gene sets from MSigDB (NES, normalized enrichment score; NOM, nominal; FDR, false discovery rate).

To elucidate the molecular pathways that TBX3 impacts to induce a transformed phenotype in hMSCs, we performed microarray analysis with RNA from TBX3 hMSCs (TBX3 #1, TBX3 #2) and EV hMSCs (EV #1, EV #2). The results identified a total of 1256 differentially expressed genes (DEGs) (903 upregulated and 353 downregulated in TBX3 hMSCs) (**Figures 9A, B****)**. Gene ontology analysis revealed that the significantly upregulated genes were enriched in biological processes related to different aspects of the cell cycle including mitosis and cell division, and the significantly downregulated genes were enriched in biological processes such as development, cell adhesion and differentiation (**Figure 9C**). Furthermore, gene set enrichment analysis (GSEA) of the DEGs showed significant differences (FDR < 0.25, NOM p-value < 0.05) in the enrichment of MSigDB Collection Hallmarks (h.all.v7.4.symbols.gmt) in TBX3 hMSCs including gene sets related to E2F targets, MYC targets, the G2/M checkpoint, mitotic spindle and DNA repair (**Figure 9D**). Analysis of the DEGs revealed that they correlated with the biological processes that TBX3 was shown to promote in this study such as cell cycle progression, migration, and invasion (**Table 1**) and KEGG analysis confirmed that the genes upregulated in TBX3 hMSCs were involved in several oncogenic signaling pathways (**Table 2**). Importantly, several of the top upregulated genes (FC > 5) were associated with sarcomagenesis (**Supplementary Table 1**) and 57 of the upregulated genes in TBX3 hMSCs were present in the CINSARC (Complexity INdex in SARComas) database which contains 67 genes associated with sarcoma aggressiveness and poor prognosis (44) (**Table 3**). Together, these results reveal the genes and pathways that TBX3 impact to promote cellular transformation in hMSCs and provide evidence of a key role for TBX3 in sarcomagenesis.

**Table 1:** TBX3 targets and selection of genes involved in cell cycle progression, migration, and invasion. Fold changes < 2 or > -2 are indicated.

Genes	Upregulated	Downregulated
**TBX3 targets**	ID1|SNAI2|TWIST1|BRAF (1,27)	CDKN2A| CDKN1C (–1,68)| CDKN1A (–1,26)
**Cell cycle progression**	CCNA2|CCNB2|CCNB1|CCND2|CCNE2|CCNF|CDK1| CDK2|CDK15|CDC6|CDC7|CDC20|CDC25B|CDC45| CDCA2|CDCA3|CDCA8|PLK1|TTK|PTTG1|MAD2L1| CHEK1|PCNA|BUB1|ORC6|MCM5|MCM3|MCM2| ID1|AURKA|AURKB|MKI67|FOXM1|MYC (1,67)	CDKN2A|CDKN2B| CDKN1C (–1,68)| CDKN1A (–1,26)
**Migration**	ZEB2|SNAI2|TWIST1|GLI3|ID1|vimentin (1,52)| CTNNB1 (1,22)	CDH6|CDH10|CDH13
**Invasion**	ADAM19|ADAM33|ADAMTS2|ADAMTS5|ADAMTS9| ADAMTS14|RUNX2|MMP16|MMP23A/B (1.69)| MMP14 (1,63)|MMP2 (1,5)|MMP9 (1,03)	TIMP1

**Table 2:** Significantly enriched cancer associated signaling pathways and genes involved identified by KEGG pathway analysis.

Signaling pathways	Genes	-Log10 (p-val)
**PI3K-Akt**	ITGB3|ITGA3|ANGPT1|CCNE2|EFNA5|CCND2|LPAR1|GNG11| IRS1|F2R|CDK2|CREB3L1|GNG2|PIK3R1|TNC|FGF5|COL6A2| KITLG	5,38
**cGMP-PKG**	KCNJ8|PDE3A|MYLK|IRS1|CREB3L1|GUCY1A2|SLC8A1| EDNRA|AGTR1|MYL9	3,79
**Rap1**	RASGRP3|ITGB3|LPAR1|EFNA5|ID1|F2R|PIK3R1|FGF5|ANGPT1| KITLG	3,04
**cAMP**	GRIN2D|F2R|GRIA3|PDE3A|PTGER3|GLI3|CREB3L1|PIK3R1| EDNRA|MYL9	2,98
**MAPK**	CACNA1H|RASGRP3|FLNC|STMN1|GADD45A|EFNA5|KITLG| CDC25B|FLNA|FGF5|ANGPT1|DUSP10	2,96
**TGF-beta**	DCN|GREM2|RBL1|THSD4|ID1|RGMB	2,61
**Phospholipase D**	PLPP3|PPAP2B|F2R|PIK3R1|AGTR1|LPAR1|KITLG	2,27
**Calcium**	CACNA1H|MYLK|PTGER3|F2R|GRIN2D|SLC8A1|EDNRA|AGTR1	2,19
**AMPK**	CCNA2|IRS1|CD36|PFKP|CREB3L1|PIK3R1	2,12
**Platelet activation**	ITGB3|PTGS1|MYLK|F2R|GUCY1A2|PIK3R1	2,06
**Metabolic pathways**	PTGS1|VDR|PPAP2B|DHFR|HYI|GPAT2|P4HA3|RRM2|RRM1| ASS1|QPRT|GPAM|PRPS1|PDE3A|PFKP|PDE7B|GUCY1A2| SYNJ2|IL4I1|PIP4K2A|CECR1|PLPP3|ST3GAL6|IDH2|TK1| ST6GALNAC3|HACD4|INPP5F|DHFRP1|AK5|TYMS	1,99
**Apelin**	EGR1|GNG11|MYLK|GNG2|SLC8A1|AGTR1	1,87
**Ras**	RASGRP3|EFNA5|GNG11|GNG2|PIK3R1|FGF5|ANGPT1|KITLG	1,76
**Wnt**	DAAM2|CCND2|DKK1|SERPINF1|FOSL1|WNT5A	1,58
**Hedgehog**	GLI3|CSNK1G1|CCND2	1,52
**Relaxin**	CLGN|CREB3L1|GNG2|GNG11|PIK3R1	1,43

**Table 3:** Significantly upregulated genes (p < 0.05) are associated with the CINSARC signature.

CINSARC signature	Gene	Fold change
**Cell cycle** (3 out of 3)	UBE2C	8,2
	FOXM1	7,63
	ASPM	3,77
**Mitosis checkpoint** (9 out of 9)	CCNA2	9,82
	CCNB2	8,78
	CCNB1	7,02
	CKS2	5,22
	CDC7	4,21
	MELK	4,04
	CDCA3	2,65
	CDC45	2,43
	CDC20	2,28
**Chromosome biogenesis** (21 out of 26)	NCAPH	3,98
	SMC2	3,17
	CHEK1	3,04
	H2AFX	3,54
	MCM2	2,85
	AURKA	2,56
	AURKB	5,97
	MAD2L1	3,62
	BUB1	2,66
	SGOL2	4,84
	PTTG1	7,72
	CENPE	2,22
	NUF2	6,15
	CDCA8	3,93
	ZWINT	2,27
	TOP2A	7,52
	BUB1B	1,78
	ESPL1	1,61
	MCM7	1,58
	CENPL	1,47
	BIRC5	1,37
**Kinesin complex** (8 out 8)	KIF11	4,71
	KIF15	2,77
	KIF23	4,06
	KIF4A	2,74
	KIF14	2,68
	KIF18A	5,49
	KIF20A	13,18
	KIF2C	2,2
**Cytokinesis** (4 out 4)	ECT2	2,26
	ANLN	17,16
	PBK	3,66
	PRC1	8,13
**Spindle and centrosome** (8 out of 12)	RRM2	4,37
	PLK4	5,74
	TPX2	8,54
	CDC6	3,59
	NEK2	2,2
	CEP55	4,15
	TTK	3,39
	FBXO5	2,29
**DNA replication and repair** (2 out of 2)	RAD51AP1	4,42
	RNASEH2A	4,88
**Other** (2 out of 3)	CDCA2	5,38
	TRIP13	3,47

## Discussion

The c-Myc and TBX3 oncoproteins are overexpressed in several sarcoma subtypes including chondrosarcoma, liposarcoma and rhabdomyosarcoma where they exert tumorigenic effects (1, 15, 17). Furthermore, while c-Myc transcriptionally activates TBX3 in these sarcomas (15, 16), whether TBX3 is a c-Myc target gene in MSCs, and whether the overexpression of TBX3 in MSCs can promote sarcomagenesis is still unknown. This study shows that TBX3 is indeed a downstream target of c-Myc in hMSCs and that endogenous TBX3 contributes to hMSC proliferation and migration. Furthermore, the overexpression of TBX3 in hMSCs promoted several hallmarks of cancer including stemness, proliferation, migration, and invasion. Together, our data suggest that the upregulation of c-Myc and consequently TBX3 may be a key event that promotes sarcomagenesis, and that this event either on its own or in combination with other oncogenic hits may transform MSCs into sarcomas.

Amplification of the c-myc gene occurs in several soft tissue and bone sarcomas and this plays an important role in their development (1). Furthermore, the ectopic overexpression of c-Myc in MSCs can promote sarcomagenesis (18, 19). However, the full repertoire of c-Myc target genes that are responsible for mediating its roles in sarcomagenesis is poorly understood. Myc is a basic/helix–loop–helix/leucine zipper (b/HLH/Zip) transcription factor that activates its target genes by binding to the canonical E-box motif CACGTG (45). Here we demonstrate that c-Myc binds to and activates the TBX3 promoter at a canonical CACGTG E-box binding site located at -701 bp. Interestingly, Willmer et al. demonstrated that in chondrosarcoma cells, c-Myc activates the TBX3 promoter through the E-box at -701 bp as well as a noncanonical E-box (GTGCAC) at -1210 bp (16). This suggests that the site(s) at which c-Myc binds the TBX3 promoter may be dependent on the cell type and levels of c-Myc. Indeed, this is consistent with reports that overexpression of c-Myc regulates its target genes through additional, previously unoccupied, canonical and noncanonical E-boxes, leading to more sustained overexpression of its targets to promote the cancer phenotype (45).

Emerging evidence suggests that transforming chromosomal abnormalities and/or mutations in MSCs may be responsible for sarcoma development, implying that MSCs may become a powerful tool to model the pathogenesis of sarcomas. For example, Shimizu et al. showed that c-Myc overexpression either on its own or in combination with loss of Ink4a-Arf in murine bone marrow derived MSCs led to the formation of osteosarcoma (19). Since our study demonstrated that c-Myc activates TBX3 and that the overexpression of TBX3 on its own in hMSCs resulted in the regulation of a set of c-Myc target genes, it is tempting to speculate that when c-Myc is amplified/overexpressed it upregulates TBX3 which then functions as one of its key mediators to transform MSCs into sarcomas.

Cancer stem cells (CSCs) comprise a subpopulation of cancer cells that drive tumor initiation and progression and TBX3 was reported to contribute to the expansion of breast CSCs and cancer stemness of ovarian and pancreatic ductal adenocarcinoma CSCs. Indeed, estrogen signaling increased the number of breast CSCs through paracrine FGF/Tbx3 signaling and silencing Tbx3 attenuated tumor sphere formation (46). Furthermore, the HOTAIR/miR‐206/TBX3 axis was shown to mediate cancer stemness of ovarian CSCs (47) and pancreatic ductal adenocarcinoma CSCs express high levels of TBX3 and sustain stemness *via* an autocrine TBX3-ACTIVIN/NODAL signaling loop (48). The results from this study show that overexpressing TBX3 in hMSCs enhances their stemness and self-renewal which correlated with a 2-fold increase in NANOG levels. NANOG is important for CSC maintenance, as it regulates the expression of other stem cell markers OCT4, SOX2, KLF4, and promotes sarcoma CSC features such as spheroid formation, anchorage-independent growth, migration and invasion (49). This raises the question as to whether NANOG is a direct target and mediator of TBX3 in enhancing stemness and self-renewal and in sarcomagenesis and future studies should investigate this. Together, these findings extend our current knowledge of the role of TBX3 in CSCs and highlight the role of TBX3 in maintaining the stem cell population that contributes to tumourigenesis and its potential as a key initiator of sarcoma.

Here we show that TBX3 overexpression in hMSCs leads to a downregulation of the p14^ARF^/MDM2/p53 tumor suppressor pathway, which could be responsible for the observed increase in cell proliferation. Under conditions of oncogenic stress, the tumor suppressor p14^ARF^ upregulates p53 expression by sequestering the p53 antagonist MDM2, thereby preventing uncontrolled cell cycle progression and proliferation (50). TBX3 was reported to repress p14^ARF^ transcription directly by binding a T-element present in the initiator or epigenetically by recruiting histone deacetylases (22, 38, 51, 52). It would therefore be worthwhile to investigate the mechanism by which TBX3 downregulates p14^ARF^ expression in hMSCs. Furthermore, we show that TBX3 promotes senescence bypass in hMSCs presumably *via* downregulation of p16^INK4a^. A report by Kumar et al. demonstrated that TBX3 can bypass senescence in primary cells and mouse embryos by forming a co-repressor complex with Coactivator of AP1 and Estrogen Receptor (CAPERα) to repress *Urothelial Cancer Associated 1* (*UCA1*) transcription which can no longer stabilize *p16^INK4a^* mRNA by sequestering Heterogeneous ribonucleoprotein A1 (HnRNP A1) (53). It would be interesting to establish whether TBX3 downregulates *p16^INK4a^* expression in hMSCs through the same mechanism reported by Kumar et al. (53). Transcriptomic analyses further demonstrated that the mRNA expression level of *CDKN2A*, encoding for both p14^ARF^ and p16^INK4a^, was also decreased by TBX3 in hMSCs and many cell cycle progression related genes including *cyclins* and *CDKs* were significantly upregulated, further confirming that TBX3 exerts a pro-proliferative effect on hMSCs *via* the downregulation and upregulation of negative and positive cell cycle regulators respectively.

TBX3 was previously shown to promote migration and invasion of several sarcoma subtypes (15, 17) and here we reveal that, when overexpressed in hMSCs, TBX3 also promotes these oncogenic processes. In sarcomas, signaling pathways including the MAPK/ERK, Wnt/β-catenin, TGF-β and PI3K/Akt, EMT transcription factors ZEB1/2, SLUG, SNAIL, and TWIST1 promote migration, invasion, and metastasis (1). Transcriptomic analyses revealed that these pathways as well as several EMT transcription factors are also upregulated in TBX3 hMSCs, and we validated overexpression of SLUG, SNAIL and TWIST1 using western blotting. These results are interesting in light of recent findings that demonstrate that TBX3 promotes breast cancer migration and invasion *via* upregulation of SLUG and TWIST1 expression (54). Furthermore, our study showed that TBX3 significantly promoted the invasion of hMSCs into collagen I matrices which corresponded with an increase in MMP2 and MMP9 levels. Our 3D spheroid invasion model resembles *in vivo* structures more closely than conventional 2D models because spheroids embedded in collagen I can naturally interact with the extracellular matrix (ECM) (55). In sarcomas, ECM production is frequently increased, resulting in a stiffer stroma with higher collagen content and a more aggressive phenotype (56). Furthermore, increased secretion of MMP2 and MMP9 correlated with metastatic potential in bone and soft tissue sarcomas (56, 57). It has been demonstrated that increased collagen I expression results in the activation of MMP2 in osteosarcoma cell lines (58), and MMP2 has been shown to promote osteosarcoma migration and invasion. Furthermore, upstream PI3K/Akt and ERK signaling pathways upregulate MMP2 and MMP9 expression, thus enabling osteosarcoma invasion and metastasis (56). Although we did not observe a FC > 2 for MMP2 (FC: 1,5; p=0,001) and MMP9 (FC: 1,03; p=0,961) mRNA expression in TBX3 hMSCs, we speculate that the observed increase in MMP2 and MMP9 protein levels is due to their activation in the ECM by proteolytic processing. Together, our study demonstrates that TBX3 promotes several cancer hallmarks in 2D- and 3D-hMSC models and future studies are required to investigate whether TBX3 alone can transform hMSCs into sarcomas *in vivo*.

Together, our study reveals that TBX3 may be a driving factor for the initiation of sarcomagenesis and thus provides additional support for the hypothesis that targeting TBX3 for anti-sarcoma treatment may be a promising approach.

## Data Availability Statement

The datasets presented in this study can be found in online repositories. The names of the repository/repositories and accession number(s) can be found below: https://www.ncbi.nlm.nih.gov/geo/, GSE183848.

## Ethics Statement

The studies involving human participants were reviewed and approved by Research Ethics Committee of the Faculty of Health Sciences, University of Pretoria (protocol number 218/2010). The patients/participants provided their written informed consent to participate in this study.

## Author Contributions

VD and SP conceived and designed the entire study while MA and MP assisted with the design and execution of selected elements. VD conducted most of the experiments, performed data analysis and wrote the manuscript. SS established the stable c-Myc overexpressing hMSCs. AN-M performed luciferase assays. CD assisted with the immunophenotype analysis of hMSCs, and MA performed the microarray experiments. SP, MA, and MP revised the manuscript. All authors contributed to the article and approved the submitted version.

## Funding

The authors were supported by grants from the South Africa Medical Research Council (SAMRC), the National Research Foundation (NRF), Cancer Association of South Africa (CANSA) and the Universities of Cape Town and Pretoria.

## Conflict of Interest

The authors declare that the research was conducted in the absence of any commercial or financial relationships that could be construed as a potential conflict of interest.

## Publisher’s Note

All claims expressed in this article are solely those of the authors and do not necessarily represent those of their affiliated organizations, or those of the publisher, the editors and the reviewers. Any product that may be evaluated in this article, or claim that may be made by its manufacturer, is not guaranteed or endorsed by the publisher.
