# Supplementary material for: The c-Myc/TBX3 Axis Promotes Cellular Transformation of Sarcoma-Initiating Cells
Source: Front Oncol. 2022 Jan 25;11:801691. doi: 10.3389/fonc.2021.801691 (PMC8821881; doi:10.3389/fonc.2021.801691)
Supplement: Supplementary file 1 [file DataSheet_1.pdf]

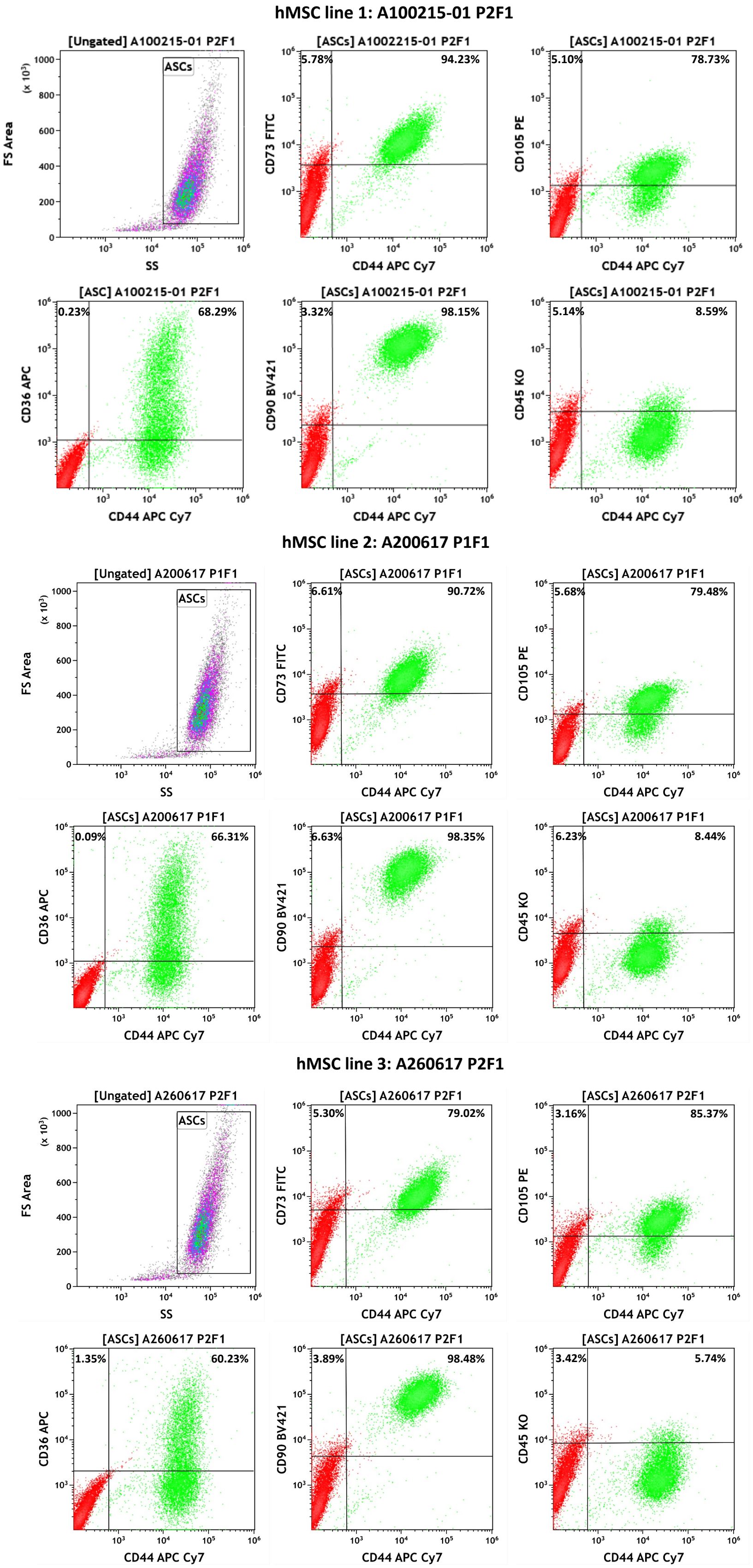

Supplementary Figure 1. Immunophenotype of hMSC lines. Phenotypic characterization of three independent hMSC (ASC) cultures (A100215-01, A200617, A260617). An unstained control was used to determine the negative/positive boundaries of the respective markers (CD73, CD105, CD44, CD36, CD90, CD45). CD44 was used as an anchor (backbone) marker. Data is displayed as overlay plots with the red population representing the unstained sample and the green population indicated the sample simultaneously stained with the various monoclonal antibodies.
