# Supplementary material for: The c-Myc/TBX3 Axis Promotes Cellular Transformation of Sarcoma-Initiating Cells
Source: Front Oncol. 2022 Jan 25;11:801691. doi: 10.3389/fonc.2021.801691 (PMC8821881; doi:10.3389/fonc.2021.801691)
Supplement: Supplementary file 2 [file DataSheet_2.pdf]

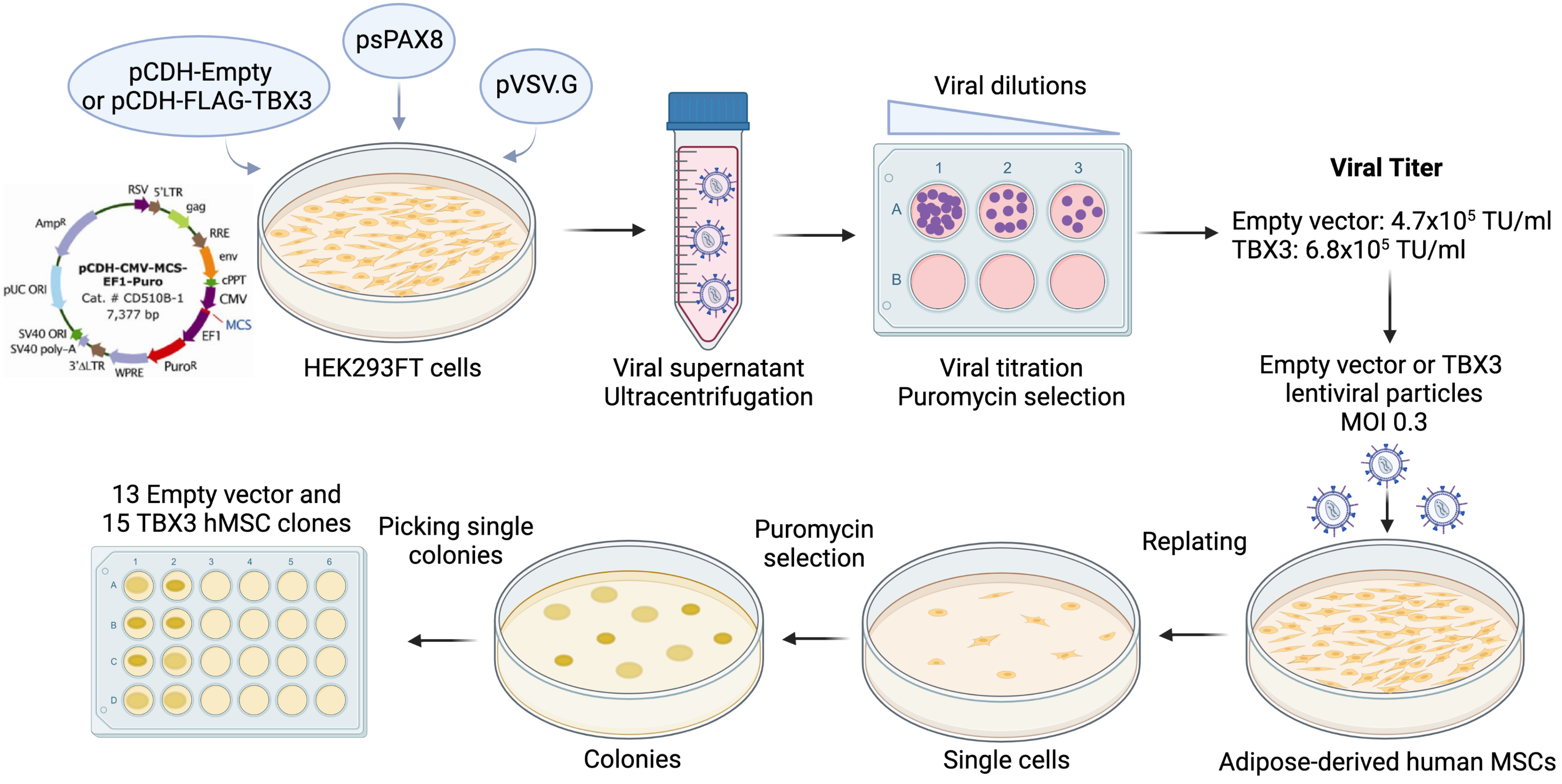

Supplementary Figure 2. Flow diagram showing lentiviral transduction method to establish Empty vector (EV) and FLAG-TBX3 hMSCs. This figure was created using biorender.com.
