# Supplementary material for: The c-Myc/TBX3 Axis Promotes Cellular Transformation of Sarcoma-Initiating Cells
Source: Front Oncol. 2022 Jan 25;11:801691. doi: 10.3389/fonc.2021.801691 (PMC8821881; doi:10.3389/fonc.2021.801691)
Supplement: Supplementary file 3 [file Table_1.docx]

**Supplementary Table 1.** Top significantly upregulated genes (FC > 5, p < 0.05) and involvement in sarcomagenesis.

| **Gene** | **Sarcoma type** | **Function** | **Reference** |
| --- | --- | --- | --- |
| HIST1H2BM | EwS | Repressed following treatment with a KDM1A inhibitor | (1) |
| KIAA0101 | Fibrosarcoma | Promotes proliferation, migration, invasion, metastasis | (2,3) |
| ZIC1 | Liposarcoma | Overexpressed; promotes proliferation and invasion, inhibits apoptosis | (4) |
| ANLN | Metastatic sarcoma,  Liposarcoma | Overexpressed; associated with poor prognosis | (5,6) |
| DEPDC1 | STS | Overexpressed; associated with high-grade, metastasis, and poor prognosis | (7) |
| KIF20A | Several STS | Overexpressed; promotes proliferation, migration, and invasion; inhibits apoptosis  Associated with poor prognosis | (8,9) |
| PLK1 | Osteosarcoma, EwS,  RMS | Overexpressed; promotes cell cycle progression | (10–12) |
| DTL | EwS | Overexpressed; promotes proliferation | (13) |
| TNC | EwS | Promotes invasion and metastasis | (14,15) |
| CCBE1 | GIST | Associated with poor prognosis; promotes angiogenesis | (16) |
| HIST1H3B | Sarcoma | Overexpressed | (17) |
| CDKN3 | Liposarcoma | Overexpressed; associated with poor prognosis | (6) |
| STMN1 | Uterine LMS,  Fibrosarcoma,  Osteosarcoma | Overexpressed; associated with poor prognosis; promotes migration, invasion, and metastasis | (18–22) |
| CENPF | STS, osteosarcoma,  synovial sarcoma | Associated with poor overall and disease-free survival  Involved in cell cycle (chromosome segregation during mitosis) and metabolism, promotes tumor growth in vivo | (8,23,24) |
| HAS2 | Fibrosarcoma, RMS | Promotes metastasis | (25–27) |
| CCNA2 | Liposarcoma | Overexpressed; associated with poor prognosis | (6,28) |
| MASP1 | Uterine LMS | Overexpressed | (29) |
| MYLK | LMS | Overexpressed | (30–32) |
| CCNB2 | LMS, STS,  Metastatic sarcoma | Overexpressed | (5,33) |
| MKI67 | Synovial sarcoma,  Uterine LMS | Overexpressed; Marker of proliferation | (18,24,34) |
| TPX2 | Several Sarcomas,  EwS,  Metastatic sarcoma,  Synovial sarcoma | Overexpressed; upregulates Aurora-A, a key regulator of mitosis; downstream target of EWS-FLI1 in EwS; promotes metastasis | (5,35–37) |
| MYOCD | LMS, Liposarcoma,  Undifferentiated sarcoma | Overexpressed; promotes migration and smooth muscle differentiation | (31,38,39) |
| UBE2C | Metastatic sarcoma,  RMS | Overexpressed | (5,27) |
| PRC1 | Metastatic sarcoma | Overexpressed | (5) |
| SLIT2 | Osteosarcoma | Overexpressed; promotes proliferation, inhibits apoptosis, contributes to the Warburg effect | (40) |
| PTTG1 | LMS, UPS, DL | Overexpressed; associated with metastasis and poor prognosis | (41) |
| HIST1H1B | Synovial sarcoma | Upregulated in metastases | (24) |
| FOXM1 | Metastatic sarcoma,  Several STS and bone sarcomas,  Synovial sarcoma | Overexpressed; promotes cell cycle progression, EMT, pluripotency, stem cell pathways | (5,42–46) |
| TOP2A | Metastatic sarcoma,  Liposarcoma,  Synovial sarcoma | Overexpressed; associated with poor prognosis | (5,24,32,47) |
| SPC25 | Metastatic sarcoma | Overexpressed | (5) |
| CCNB1 | Metastatic sarcoma,  Liposarcoma, LMS,  RMS | Overexpressed; associated with poor prognosis | (5,6,33,48) |
| HJURP | Synovial sarcoma,  osteosarcoma | Associated with metastasis and poor prognosis | (37,49) |
| RACGAP1 | Uterine Carcinosarcoma | Overexpressed; promotes invasion and metastasis | (50) |
| NUF2 | Metastatic sarcoma | Overexpressed | (5) |
| NCAPG | Synovial sarcoma,  STS | Associated with metastasis and poor prognosis | (37,51) |
| AURKB | Liposarcoma,  Synovial sarcoma,  RMS, osteosarcoma | Overexpressed; associated with poor prognosis; promotes cell cycle progression,  migration, and invasion | (6,24,48,52,53) |
| NDC80 | Osteosarcoma,  MFS, Liposarcoma,  LMS | Overexpressed; associated with poor prognosis | (54) |
| PLK4 | Metastatic sarcoma, sarcoma | Overexpressed; promotes tumor formation | (5,55) |
| CNN1 | LMS | Overexpressed | (31,32) |
| CDK1 | RMS | Overexpressed; associated with poor prognosis | (48,51) |
| KIF18A | Metastatic sarcoma,  Synovial sarcoma | Overexpressed | (5,56) |
| CDCA2 | Metastatic sarcoma,  Synovial sarcoma | Overexpressed; associated with poor prognosis | (5,57) |
| CKS2 | Uterine LMS | Overexpressed; associated with poor prognosis | (58) |
| SMYD3 | Fibrosarcoma | Promotes migration and invasion | (59) |

Abbreviations: STS: Soft tissue sarcoma, LMS: Leiomyosarcoma, EwS: Ewing’s sarcoma, GIST: Gastrointestinal stromal tumor, DL: Dedifferentiated liposarcoma, UPS: Undifferentiated pleomorphic sarcoma, RMS: Rhabdomyosarcoma, MFS: Myxofibrosarcoma

**Supplementary references**

1. Pishas KI, Drenberg CD, Taslim C, Theisen ER, Johnson KM, Saund RS, Pop IL, Crompton BD, Lawlor ER, Tirode F, et al. Therapeutic Targeting of KDM1A/LSD1 in Ewing Sarcoma with SP-2509 Engages the Endoplasmic Reticulum Stress Response. *Mol Cancer Ther* (2018) **17**:1902–1916. doi:10.1158/1535-7163.MCT-18-0373

2. Samantarrai D, Mallick B. miR-429 inhibits metastasis by targeting KIAA0101 in Soft Tissue Sarcoma. *Exp Cell Res* (2017) **357**:33–39. doi:10.1016/j.yexcr.2017.04.017

3. Jain N, Roy J, Das B, Mallick B. miR‐197‐5p inhibits sarcomagenesis and induces cellular senescence via repression of KIAA0101. *Mol Carcinog* (2019) **58**:1376–1388. doi:10.1002/mc.23021

4. Brill E, Gobble R, Angeles C, Lagos-Quintana M, Crago A, Laxa B, DeCarolis P, Zhang L, Antonescu C, Socci ND, et al. ZIC1 Overexpression Is Oncogenic in Liposarcoma. *Cancer Res* (2010) **70**:6891–6901. doi:10.1158/0008-5472.CAN-10-0745

5. Jemaà M, Abdallah S, Lledo G, Perrot G, Lesluyes T, Teyssier C, Roux P, van Dijk J, Chibon F, Abrieu A, et al. Heterogeneity in sarcoma cell lines reveals enhanced motility of tetraploid versus diploid cells. *Oncotarget* (2017) **8**:16669–16689. doi:10.18632/oncotarget.14291

6. Skubitz KM, Skubitz AP, Xu WW, Luo X, Lagarde P, Coindre J-M, Chibon F. Gene expression identifies heterogeneity of metastatic behavior among high-grade non-translocation associated soft tissue sarcomas. *J Transl Med* (2014) **12**:176. doi:10.1186/1479-5876-12-176

7. Pollino S, Benassi MS, Pazzaglia L, Conti A, Bertani N, Righi A, Piccinni-Leopardi M, Picci P, Perris R. Prognostic role of XTP1/DEPDC1B and SDP35/DEPDC1A in high grade soft-tissue sarcomas. *Histol Histopathol* (2018) **33**:597–608. doi:10.14670/HH-11-959

8. Zhu Z, Jin Z, Deng Y, Wei L, Yuan X, Zhang M, Sun D. Co-expression Network Analysis Identifies Four Hub Genes Associated With Prognosis in Soft Tissue Sarcoma. *Front Genet* (2019) **10**: doi:10.3389/fgene.2019.00037

9. Zhu Z, Jin Z, Zhang H, Zhang M, Sun D. Knockdown of Kif20a inhibits growth of tumors in soft tissue sarcoma in vitro and in vivo. *J Cancer* (2020) **11**:5088–5098. doi:10.7150/jca.44777

10. Chou Y-S, Yen C-C, Chen W-M, Lin Y-C, Wen Y-S, KE W-T, Wang J-Y, Liu C-Y, Yang M-H, Chen T-H, et al. Cytotoxic mechanism of PLK1 inhibitor GSK461364 against osteosarcoma: Mitotic arrest, apoptosis, cellular senescence, and synergistic effect with paclitaxel. *Int J Oncol* (2016) **48**:1187–1194. doi:10.3892/ijo.2016.3352

11. Hu K, Lee C, Qiu D, Fotovati A, Davies A, Abu-Ali S, Wai D, Lawlor ER, Triche TJ, Pallen CJ, et al. Small interfering RNA library screen of human kinases and phosphatases identifies polo-like kinase 1 as a promising new target for the treatment of pediatric rhabdomyosarcomas. *Mol Cancer Ther* (2009) **8**:3024–3035. doi:10.1158/1535-7163.MCT-09-0365

12. Weiß LM, Hugle M, Romero S, Fulda S. Synergistic induction of apoptosis by a polo-like kinase 1 inhibitor and microtubule-interfering drugs in Ewing sarcoma cells. *Int J Cancer* (2016) **138**:497–506. doi:10.1002/ijc.29725

13. Mackintosh C, Ordóñez JL, García-Domínguez DJ, Sevillano V, Llombart-Bosch A, Szuhai K, Scotlandi K, Alberghini M, Sciot R, Sinnaeve F, et al. 1q gain and CDT2 overexpression underlie an aggressive and highly proliferative form of Ewing sarcoma. *Oncogene* (2012) **31**:1287–1298. doi:10.1038/onc.2011.317

14. Hawkins AG, Julian CM, Konzen S, Treichel S, Lawlor ER, Bailey KM. Microenvironmental Factors Drive Tenascin C and Src Cooperation to Promote Invadopodia Formation in Ewing Sarcoma. *Neoplasia* (2019) **21**:1063–1072. doi:10.1016/j.neo.2019.08.007

15. He S, Huang Q, Hu J, Li L, Xiao Y, Yu H, Han Z, Wang T, Zhou W, Wei H, et al. EWS-FLI1-mediated tenascin-C expression promotes tumour progression by targeting MALAT1 through integrin α5β1-mediated YAP activation in Ewing sarcoma. *Br J Cancer* (2019) **121**:922–933. doi:10.1038/s41416-019-0608-1

16. Tian G-A, Zhu C-C, Zhang X-X, Zhu L, Yang X-M, Jiang S-H, Li R-K, Tu L, Wang Y, Zhuang C, et al. CCBE1 promotes GIST development through enhancing angiogenesis and mediating resistance to imatinib. *Sci Rep* (2016) **6**:31071. doi:10.1038/srep31071

17. Ohshima K, Hatakeyama K, Nagashima T, Watanabe Y, Kanto K, Doi Y, Ide T, Shimoda Y, Tanabe T, Ohnami S, et al. Integrated analysis of gene expression and copy number identified potential cancer driver genes with amplification-dependent overexpression in 1,454 solid tumors. *Sci Rep* (2017) **7**:641. doi:10.1038/s41598-017-00219-3

18. Hu X, Zhang H, Zheng X, Lin Z, Feng G, Chen Y, Pan Q, Ni F. STMN1 and MKI67 Are Upregulated in Uterine Leiomyosarcoma and Are Potential Biomarkers for its Diagnosis. *Med Sci Monit* (2020) **26**: doi:10.12659/MSM.923749

19. Baldassarre G, Belletti B, Nicoloso MS, Schiappacassi M, Vecchione A, Spessotto P, Morrione A, Canzonieri V, Colombatti A. p27Kip1-stathmin interaction influences sarcoma cell migration and invasion. *Cancer Cell* (2005) **7**:51–63. doi:10.1016/j.ccr.2004.11.025

20. Liu J, Li J, Wang K, Liu H, Sun J, Zhao X, Yu Y, Qiao Y, Wu Y, Zhang X, et al. Aberrantly high activation of a FoxM1–STMN1 axis contributes to progression and tumorigenesis in FoxM1-driven cancers. *Signal Transduct Target Ther* (2021) **6**:42. doi:10.1038/s41392-020-00396-0

21. Belletti B, Nicoloso MS, Schiappacassi M, Berton S, Lovat F, Wolf K, Canzonieri V, D’Andrea S, Zucchetto A, Friedl P, et al. Stathmin Activity Influences Sarcoma Cell Shape, Motility, and Metastatic Potential. *Mol Biol Cell* (2008) **19**:2003–2013. doi:10.1091/mbc.e07-09-0894

22. Wang Z, He R, Xia H, Wei Y, Wu S. Knockdown of STMN1 enhances osteosarcoma cell chemosensitivity through inhibition of autophagy. *Oncol Lett* (2017) **13**:3465–3470. doi:10.3892/ol.2017.5941

23. Zou P-A, Yang Z-X, Wang X, Tao Z-W. Upregulation of CENPF is linked to aggressive features of osteosarcoma. *Oncol Lett* (2021) **22**:648. doi:10.3892/ol.2021.12909

24. Song Y, Liu X, Wang F, Wang X, Cheng G, Peng C. Identification of Metastasis-Associated Biomarkers in Synovial Sarcoma Using Bioinformatics Analysis. *Front Genet* (2020) **11**: doi:10.3389/fgene.2020.530892

25. Čermák V, Kosla J, Plachý J, Trejbalová K, Hejnar J, Dvořák M. The transcription factor EGR1 regulates metastatic potential of v-src transformed sarcoma cells. *Cell Mol Life Sci* (2010) **67**:3557–3568. doi:10.1007/s00018-010-0395-6

26. Itano N, Sawai T, Atsumi F, Miyaishi O, Taniguchi S, Kannagi R, Hamaguchi M, Kimata K. Selective Expression and Functional Characteristics of Three Mammalian Hyaluronan Synthases in Oncogenic Malignant Transformation. *J Biol Chem* (2004) **279**:18679–18687. doi:10.1074/jbc.M313178200

27. Würtemberger J, Tchessalova D, Regina C, Bauer C, Schneider M, Wagers AJ, Hettmer S. Growth inhibition associated with disruption of the actin cytoskeleton by Latrunculin A in rhabdomyosarcoma cells. *PLoS One* (2020) **15**:e0238572. doi:10.1371/journal.pone.0238572

28. Francis P, Namløs H, Müller C, Edén P, Fernebro J, Berner J-M, Bjerkehagen B, Åkerman M, Bendahl P-O, Isinger A, et al. Diagnostic and prognostic gene expression signatures in 177 soft tissue sarcomas: hypoxia-induced transcription profile signifies metastatic potential. *BMC Genomics* (2007) **8**:73. doi:10.1186/1471-2164-8-73

29. Davidson B, Abeler VM, Førsund M, Holth A, Yang Y, Kobayashi Y, Chen L, Kristensen GB, Shih I-M, Wang T-L. Gene expression signatures of primary and metastatic uterine leiomyosarcoma. *Hum Pathol* (2014) **45**:691–700. doi:10.1016/j.humpath.2013.11.003

30. Hoang NT, Acevedo LA, Mann MJ, Tolani B. A review of soft-tissue sarcomas: Translation of biological advances into treatment measures. *Cancer Manag Res* (2018) **10**:1089–1114. doi:10.2147/CMAR.S159641

31. Villacis RAR, Silveira SM, Barros-Filho MC, Marchi FA, Domingues MAC, Scapulatempo-Neto C, Aguiar S, Lopes A, Cunha IW, Rogatto SR. Gene Expression Profiling in Leiomyosarcomas and Undifferentiated Pleomorphic Sarcomas: SRC as a New Diagnostic Marker. *PLoS One* (2014) **9**:e102281. doi:10.1371/journal.pone.0102281

32. Baird K, Davis S, Antonescu CR, Harper UL, Walker RL, Chen Y, Glatfelter AA, Duray PH, Meltzer PS. Gene Expression Profiling of Human Sarcomas: Insights into Sarcoma Biology. *Cancer Res* (2005) **65**:9226–9235. doi:10.1158/0008-5472.CAN-05-1699

33. Schachtschneider KM, Liu Y, Mäkeläinen S, Madsen O, Rund LA, Groenen MAM, Schook LB. Oncopig Soft-Tissue Sarcomas Recapitulate Key Transcriptional Features of Human Sarcomas. *Sci Rep* (2017) **7**:2624. doi:10.1038/s41598-017-02912-9

34. Krsková L, Kalinová M, Břízová H, Mrhalová M, Sumerauer D, Kodet R. Molecular and immunohistochemical analyses of BCL2, KI-67, and cyclin D1 expression in synovial sarcoma. *Cancer Genet Cytogenet* (2009) **193**:1–8. doi:10.1016/j.cancergencyto.2009.03.008

35. Asteriti IA, Rensen WM, Lindon C, Lavia P, Guarguaglini G. The Aurora-A/TPX2 complex: A novel oncogenic holoenzyme? *Biochim Biophys Acta - Rev Cancer* (2010) **1806**:230–239. doi:10.1016/j.bbcan.2010.08.001

36. Wates RJ, Ma Y, Crow J, Samuel G, Godwin AK. Abstract 4141: EWS-FLI regulates mitotic kinesins in Ewing sarcoma family of tumors. in *Tumor Biology* (American Association for Cancer Research), 4141–4141. doi:10.1158/1538-7445.AM2018-4141

37. Wu H, Zhang B, Zhao J, Zhao Y, Ma X, Feng H. Weighted Gene Co-Expression Network Analysis Identifies Five Hub Genes Associated with Metastasis in Synovial Sarcoma. *Comb Chem High Throughput Screen* (2021) **24**: doi:10.2174/1386207324666210628112429

38. Abeshouse A, Adebamowo C, Adebamowo SN, Akbani R, Akeredolu T, Ally A, Anderson ML, Anur P, Appelbaum EL, Armenia J, et al. Comprehensive and Integrated Genomic Characterization of Adult Soft Tissue Sarcomas. *Cell* (2017) **171**:950-965.e28. doi:10.1016/j.cell.2017.10.014

39. Perot G, Derre J, Coindre J-M, Tirode F, Lucchesi C, Mariani O, Gibault L, Guillou L, Terrier P, Aurias A. Strong Smooth Muscle Differentiation Is Dependent on Myocardin Gene Amplification in Most Human Retroperitoneal Leiomyosarcomas. *Cancer Res* (2009) **69**:2269–2278. doi:10.1158/0008-5472.CAN-08-1443

40. Zhao S-J, Shen Y-F, Li Q, He Y-J, Zhang Y-K, Hu L-P, Jiang Y-Q, Xu N-W, Wang Y-J, Li J, et al. SLIT2/ROBO1 axis contributes to the Warburg effect in osteosarcoma through activation of SRC/ERK/c-MYC/PFKFB2 pathway. *Cell Death Dis* (2018) **9**:390. doi:10.1038/s41419-018-0419-y

41. Li Y-L, Gao Y-L, Niu X-L, Wu Y-T, Du Y-M, Tang M-S, Li J-Y, Guan X-H, Song B. Identification of Subtype-Specific Metastasis-Related Genetic Signatures in Sarcoma. *Front Oncol* (2020) **10**: doi:10.3389/fonc.2020.544956

42. Kelleher FC, O’Sullivan H. FOXM1 in sarcoma: role in cell cycle, pluripotency genes and stem cell pathways. *Oncotarget* (2016) **7**:42792–42804. doi:10.18632/oncotarget.8669

43. Eisinger-Mathason TSK, Mucaj V, Biju KM, Nakazawa MS, Gohil M, Cash TP, Yoon SS, Skuli N, Park KM, Gerecht S, et al. Deregulation of the Hippo pathway in soft-tissue sarcoma promotes FOXM1 expression and tumorigenesis. *Proc Natl Acad Sci* (2015) **112**:E3402–E3411. doi:10.1073/pnas.1420005112

44. Ke X-Y, Chen Y, Tham VY-Y, Lin RY-T, Dakle P, Nacro K, Puhaindran ME, Houghton P, Pang A, Lee VK, et al. MNK1 and MNK2 enforce expression of E2F1, FOXM1, and WEE1 to drive soft tissue sarcoma. *Oncogene* (2021) **40**:1851–1867. doi:10.1038/s41388-021-01661-4

45. Maekawa A, Kohashi K, Kuda M, Iura K, Ishii T, Endo M, Nakatsura T, Iwamoto Y, Oda Y. Prognostic significance of FOXM1 expression and antitumor effect of FOXM1 inhibition in synovial sarcomas. *BMC Cancer* (2016) **16**:511. doi:10.1186/s12885-016-2542-4

46. Christensen L, Joo J, Lee S, Wai D, Triche TJ, May WA. FOXM1 Is an Oncogenic Mediator in Ewing Sarcoma. *PLoS One* (2013) **8**:e54556. doi:10.1371/journal.pone.0054556

47. da Cunha IW, De Brot L, Carvalho KC, Rocha RM, Fregnani JH, Falzoni R, de Oliveira Ferreira F, Júnior SA, Lopes A, Muto NH, et al. Prognostication of Soft Tissue Sarcomas Based on Chromosome 17q Gene and Protein Status: Evaluation of TOP2A, HER-2/neu, and Survivin. *Ann Surg Oncol* (2012) **19**:1790–1799. doi:10.1245/s10434-011-2184-3

48. Li Q, Zhang L, Jiang J, Zhang Y, Wang X, Zhang Q, Wang Y, Liu C, Li F. CDK1 and CCNB1 as potential diagnostic markers of rhabdomyosarcoma: validation following bioinformatics analysis. *BMC Med Genomics* (2019) **12**:198. doi:10.1186/s12920-019-0645-x

49. Li H, Liu H, Zhang Z, Xu X, Kan J, Dai S, Yang Y, Li Y. HJURP overexpression indicates unfavorable prognosis in osteosarcoma. *Int J Clin Exp Med* (2017) **10**:1145–1150.

50. Mi S, Lin M, Brouwer-Visser J, Heim J, Smotkin D, Hebert T, Gunter MJ, Goldberg GL, Zheng D, Huang GS. RNA-seq Identification of RACGAP1 as a Metastatic Driver in Uterine Carcinosarcoma. *Clin Cancer Res* (2016) **22**:4676–4686. doi:10.1158/1078-0432.CCR-15-2116

51. Lu S, Sun C, Chen H, Zhang C, Li W, Wu L, Zhu J, Sun F, Huang J, Wang J, et al. Bioinformatics Analysis and Validation Identify CDK1 and MAD2L1 as Prognostic Markers of Rhabdomyosarcoma. *Cancer Manag Res* (2020) **Volume 12**:12123–12136. doi:10.2147/CMAR.S265779

52. Mattei JC, Bouvier-Labit C, Barets D, Macagno N, Chocry M, Chibon F, Morando P, Rochwerger RA, Duffaud F, Olschwang S, et al. Pan Aurora Kinase Inhibitor: A Promising Targeted-Therapy in Dedifferentiated Liposarcomas With Differential Efficiency Depending on Sarcoma Molecular Profile. *Cancers (Basel)* (2020) **12**:583. doi:10.3390/cancers12030583

53. Zhu XP, Liu ZL, Peng AF, Zhou YF, Long XH, Luo QF, Huang SH, Shu Y. Inhibition of Aurora-B suppresses osteosarcoma cell migration and invasion. *Exp Ther Med* (2014) **7**:560–564. doi:10.3892/etm.2014.1491

54. Xu B, Wu D-P, Xie R-T, Liu L-G, Yan X-B. Elevated NDC80 expression is associated with poor prognosis in osteosarcoma patients. *Eur Rev Med Pharmacol Sci* (2017) **21**:2045–2053. Available at: http://www.ncbi.nlm.nih.gov/pubmed/28537682

55. Coelho PA, Bury L, Shahbazi MN, Liakath-Ali K, Tate PH, Wormald S, Hindley CJ, Huch M, Archer J, Skarnes WC, et al. Over-expression of Plk4 induces centrosome amplification, loss of primary cilia and associated tissue hyperplasia in the mouse. *Open Biol* (2015) **5**:150209. doi:10.1098/rsob.150209

56. Przybyl J, Sciot R, Wozniak A, Schöffski P, Vanspauwen V, Samson I, Siedlecki JA, Rutkowski P, Debiec-Rychter M. Metastatic potential is determined early in synovial sarcoma development and reflected by tumor molecular features. *Int J Biochem Cell Biol* (2014) **53**:505–513. doi:10.1016/j.biocel.2014.05.006

57. Lagarde P, Przybyl J, Brulard C, Pérot G, Pierron G, Delattre O, Sciot R, Wozniak A, Schöffski P, Terrier P, et al. Chromosome Instability Accounts for Reverse Metastatic Outcomes of Pediatric and Adult Synovial Sarcomas. *J Clin Oncol* (2013) **31**:608–615. doi:10.1200/JCO.2012.46.0147

58. Deng Y, Han Q, Mei S, Li H, Yang F, Wang J, Ge S, Jing X, Xu H, Zhang T. Cyclin‑dependent kinase subunit 2 overexpression promotes tumor progression and predicts poor prognosis in uterine leiomyosarcoma. *Oncol Lett* (2019) doi:10.3892/ol.2019.10668

59. Cock-Rada AM, Medjkane S, Janski N, Yousfi N, Perichon M, Chaussepied M, Chluba J, Langsley G, Weitzman JB. SMYD3 Promotes Cancer Invasion by Epigenetic Upregulation of the Metalloproteinase MMP-9. *Cancer Res* (2012) **72**:810–820. doi:10.1158/0008-5472.CAN-11-1052
